# Cognitive and Mental Health Profiles of Binge-Eating Adults with and Without Comorbid Addictive Behaviors

**DOI:** 10.3390/healthcare13131524

**Published:** 2025-06-26

**Authors:** Jake Jeong, Jungwon Jang, Giho Jeon, Kwangyeol Baek

**Affiliations:** 1 Department of Information Convergence Engineering, Pusan National University, Busan 50612, Republic of Korea; mekire@pusan.ac.kr (J.J.); joannajang98@pusan.ac.kr (J.J.); ghjeon854@pusan.ac.kr (G.J.); 2 Center for Artificial Intelligence Research, Pusan National University, Busan 46241, Republic of Korea; 3 School of Biomedical Convergence Engineering, Pusan National University, Busan 50612, Republic of Korea

**Keywords:** binge eating, addiction, comorbid, impulsivity, self-control, emotion regulation, depression, eating disorder

## Abstract

**Background**: Binge eating is a disordered eating behavior implicated in eating disorders such as binge eating disorder (BED) and bulimia nervosa; it significantly affects an individual’s physical and mental health. Recent studies suggest shared neurobiological mechanisms between binge eating and addictive behaviors. Comorbid addiction (e.g., substance use disorders and behavioral addictions) is also frequently reported in binge-eating patients. However, it is still unclear whether binge-eating individuals with comorbid addictions differ in their cognitive and mental health characteristics from those without comorbid addictions. **Objectives**: The present study aimed to examine the cognitive and mental health profiles of binge-eating individuals with and without co-occurring addictions. We hypothesized that binge-eating individuals with comorbid addictions would show greater impairments in impulsivity and self-control, as well as elevated depression and emotion dysregulation. **Methods**: In the present study, we assessed psychometric scales on various cognitive and mental health domains (e.g., impulsivity, behavioral inhibition, self-control, emotion regulation, mood, and anxiety) across 30 binge-eating individuals with co-occurring addictive behaviors (i.e., alcohol, nicotine, gambling, and video games), 32 binge-eating individuals without addiction, and 180 healthy control subjects with neither binge-eating tendencies nor addiction. **Results**: Both binge-eating groups showed a significant increase in punishment sensitivity, perceived stress, and state/trait anxiety compared to healthy controls, but there was no difference between the two binge-eating groups. Higher impulsivity and lower self-control were observed in both binge-eating groups to a significantly greater degree in the group with comorbid addiction. Notably, significantly increased depression and impaired emotion regulation (reduced use of cognitive reappraisal) were observed only in the binge-eating group with comorbid addiction when compared to the healthy controls. **Conclusions**: Our findings demonstrated the commonalities and differences in binge-eating populations with and without comorbid addiction. It will help to elucidate cognitive and mental health aspects of comorbid addiction in the binge-eating population and to develop more tailored diagnoses and treatments.

## 1. Introduction

Binge eating is a disordered eating behavior characterized by eating a significantly larger amount of food than typical in a discrete period of time (e.g., within 2 h) and the sense of a lack of control over eating during the episode [1], which makes it different from just overeating. Binge eating first gained clinical attention as a distinct eating pattern among obese individuals and was initially observed by Stunkard in 1959 [2]. Subsequently, binge eating has been established as a core symptom in eating disorders such as bulimia nervosa (BN) and binge eating disorder (BED).

In recent years, binge eating has been rapidly increasing among the population and poses a significant clinical risk to physical and mental well-being. For example, the prevalence of binge eating in Australia increased from 2.7% in 1998 to 13.0% by 2015, and the binge-eating population demonstrated a reduced health-related quality of life and increased days out of role [3]. A recent study in Brazil reported that the prevalence of recurrent binge eating was 6.2%, the prevalence of BED was 1.4%, and the prevalence of BN was 0.7% [4]. In this study, individuals with recurrent binge eating tendencies exhibited an increased risk of depression, anxiety, and attention deficit/hyperactivity disorder, as well as a reduced quality of life in relation to their physical and mental health. From the World Mental Healthy Surveys data, the lifetime prevalence rate of BED and BN was estimated as 1.9% and 1.0% on average [5]. Individuals with BED or BN showed a significantly higher risk of morbid obesity, other mental disorders, diabetes, hypertension, and chronic pain. In conclusion, binge eating (and related eating disorders) is a significant problem affecting physical and mental health in the present day.

The psychopathological and neurobiological mechanisms of binge eating are still under investigation, but recent studies have identified similarities with substance-related and addictive disorders [6,7,8,9]. From a reward system perspective, both animal and human studies have reported dysregulation in dopaminergic and opioid pathways that are central to compulsive reward-seeking behavior. Animal models of binge eating have shown dysfunctional dopaminergic transmission and a heightened sensitivity to palatable food cues, reflecting mechanisms similar to those observed in substance-related and addictive disorders [7]. Human neuroimaging studies have also revealed hyperactivation in striatal circuits associated with reward anticipation and valuation in individuals with binge eating tendencies and obesity [7,8,9]. Specifically, the ventral striatum has been identified as a potential biological marker, implicated in reward processing in both BED and behavioral addictions such as gambling disorder and internet gaming disorder [10]. These alterations in reward processing have been linked to increased impulsivity and craving, which may underlie both binge eating and addictive behaviors.

In contrast, impairments in cognitive control systems have also been implicated with binge eating. These include a reduced activity in prefrontal regions, such as the dorsolateral prefrontal cortex and anterior cingulate cortex, which are critical for executive functioning, response inhibition, and self-regulation [8,9]. Such deficits are consistent with the behavioral symptoms observed in binge eating—overeating larger amounts than intended, loss of control, and persistent binging despite negative consequences—all of which parallel the diagnostic criteria for substance-related and addictive disorders [9]. For example, impaired inhibitory control and elevated impulsivity were observed in patients with bulimic spectrum eating disorder, as well as those with gambling disorder [11]. Together, these converging lines of evidence support the view that binge eating may involve both heightened reward sensitivity and diminished cognitive control, reflecting a neurobehavioral profile frequently observed in addictive behaviors.

Despite accumulating evidence of shared neurobiological mechanisms between binge eating and addictive behaviors, there are limited studies of binge-eating individuals in relation to comorbid substance-related and addictive disorders. Most epidemiological studies reported the prevalence of comorbid substance use disorder in BED patients, which showed substance use disorders as one of the most frequently observed comorbid psychiatric disorders associated with BED [12]. For example, Peterson and her colleagues tried subtyping BED into BED subjects with and without substance use disorder (*n* = 39 and 45, respectively), and they found more binge-eating episodes and higher impulsivity traits in BED subjects with substance addiction [13]. In a later study, Becker and Grilo identified four subgroups in 347 BED patients (34 subjects with co-occurring substance use disorder, 129 with mood disorder, 60 with both substance use disorder and mood disorders, and 124 with neither of them) [14]. Intriguingly, the 34 BED subjects with substance addiction were not different from the group with only BED in personality traits, but the groups with comorbid mood disorders showed increased depression symptoms and lower self-esteem. However, these studies are not explicitly designed to contrast binge-eating populations with and without comorbid substance-related and addictive disorders, and insufficient attention has been paid to cognitive aspects related to addictive behaviors (e.g., motivational systems, impulsivity, self-control, emotion regulation, perceived stress, etc.). Another limitation of previous work is the narrow focus on substance-related disorders, with behavioral addictions such as gambling and gaming disorders often being overlooked despite their formal recognition in current diagnostic frameworks. In this context, a recent meta-analysis confirmed the moderate prevalence of behavioral addictions in individuals with eating disorders, highlighting the clinical relevance of such comorbidities [15]. Farstad and von Ranson [16] further found that women at risk for both binge eating and problem gambling reported significantly greater overall difficulties in emotion regulation, as well as heightened positive and negative urgency, compared to those with either condition alone. These findings point to a need for further research on the cognitive and mental health profiles of individuals with binge eating tendencies who also exhibit substance-related or behavioral addictions.

This study aimed to compare binge-eating individuals with and without co-occurring addictive behaviors, focusing on exploring the psychopathological characteristics of the comorbid group in binge-eating populations. We also compared both binge-eating populations with age-matched healthy controls to identify cognitive and mental health characteristics linked to binge eating. We hypothesized that increased impulsivity and decreased self-control might be associated with binge eating, particularly more strongly in individuals with comorbid addictions. In addition, we expected that individuals with binge eating tendencies—especially those with co-occurring addictions—would report higher levels of stress, anxiety, and depression, as well as showing greater impairment in emotion regulation strategies, such as the reduced use of cognitive reappraisal, compared to healthy controls.

## 2. Materials and Methods

### 2.1. Participants

**Figure 1:**
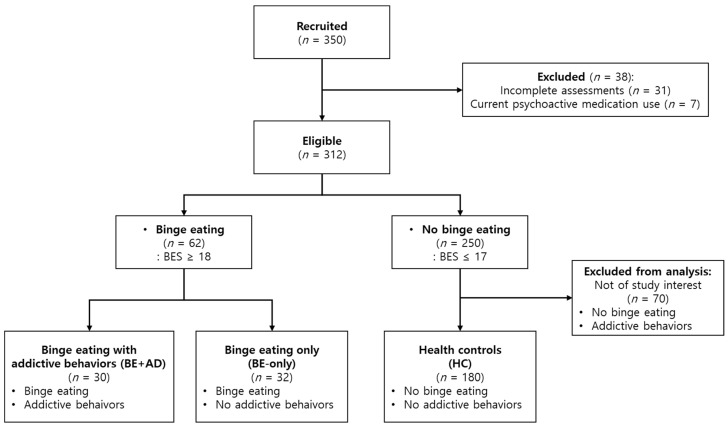
Study participant recruitment and group allocation process.

This study was conducted with the approval of the Institutional Review Board (IRB) of Pusan National University (IRB approval date: 24 November 2021). We recruited participants from the Korean adult population, aged 19 to 59, who had no history of mental disorder diagnoses other than sleep disorders. We used online advertisements posted on the internet bulletin boards of seven local universities, local community mobile apps, and SNS messengers. Participants were recruited via online advertisements titled “Online Behavioral Survey Research Participants Wanted for Addiction Proneness.” The advertisements listed several domains of addictive behaviors, including in relation to food, alcohol, nicotine, gambling, gaming, etc., which may have led to a higher proportion of respondents with pre-existing concerns about these behaviors (see Supplementary Information for full text). A total of 350 participants were initially recruited between May and June 2022 (Figure 1). The exclusion criteria were as follows: (1) incomplete assessments, (2) inconsistent responses, and (3) positive responses regarding illegal drug use or misuse of psychoactive medications. A total of 38 subjects were excluded, resulting in a total of 312 participants for data analysis. Among the 312 participants, 195 subjects were females (62.5%). As concerns age distribution, there were 196 participants of 29 years old or younger (62.8%), 68 participants in their 30s (21.8%), 29 participants in their 40s (9.3%), and 19 participants in their 50s (6.1%).

### 2.2. Screening Tools for Binge Eating

The Binge Eating Scale (BES) is a widely used 16-item self-administered screening measure of binge eating [17]. Each item is assigned a score ranging from 0 to 2 points or from 0 to 3 points based on its respective weights. Total scores below 18 points indicate ‘no binge eating,’ while scores between 18 and 26 points signify a ‘moderate level of binge eating.’ Scores of 27 or higher indicate a ‘severe level of binge eating.’ In this study, scores equal to or exceeding 18 were considered indicative of the presence of BED. The internal consistency (Cronbach’s α) in the Korean validation study was determined to be 0.84 [18], while it was determined to be 0.91 in this study.

### 2.3. Screening Tools for Addictions

All addiction-related scales used in this study (see below) served as validated screening instruments. These tools were employed to identify individuals at an elevated risk for clinically meaningful patterns of substance-related or behavioral addictions, rather than to establish formal clinical diagnoses.

#### 2.3.1. Alcohol

The Alcohol Use Disorders Identification Test (AUDIT) was used to assess alcohol addiction [19]. The scale consists of 10 items, with responses ranging from 0 to 4 points on a 5-point Likert scale. The clinical cut-off for diagnosis is set at 12 points, and scores equal to or above 12 are indicative of alcohol use disorder (AUD). The internal consistency (Cronbach’s α) in the Korean validation study was reported as 0.92 [20], while it was reported to be 0.87 in this study.

#### 2.3.2. Nicotine

The Fagerstrom Test for Nicotine Dependence (FTND) was used to examine nicotine dependence [21]. The test consists of six items, with two items scored on a scale of 0 to 3 points, and the remaining items using a dichotomous ‘yes/no’ response. In this study, we utilized a clinical cut-off of 7 or higher. The internal consistency (Cronbach’s α) in the Korean validation study was reported as 0.69 [22], while it was reported as 0.75 in this study.

#### 2.3.3. Video Games

The Clinical Video Game Addiction Test 2.0 (C-VAT 2.0) assesses video game addiction [23]. The test consists of 11 items, using a binary ‘yes/no’ response (1 point for ‘yes’; 0 points for ‘no’). A total score of 6 points or higher indicates the presence of video game addiction. The internal consistency (Cronbach’s α) in the Korean validation study was reported as 0.94 [24], while it was reported as 0.92 in this study.

#### 2.3.4. Gambling

The Canadian Problem Gambling Index (CPGI) measured problem gambling behaviors [25]. The index comprises 9 items, using a 4-point Likert scale ranging from 0 to 4 points. Scores of 0 indicate ‘non-problem gambling’, scores between 1 and 2 indicate ‘low-risk gambling’, scores between 3 and 7 indicate ‘moderate-risk gambling’, and scores of 8 or higher are categorized as ‘problem gambling’. A cut-off score of 8 or higher was utilized in this study. The internal consistency (Cronbach’s α) in the Korean validation study was reported as 0.86 [26], while it was reported as 0.93 in this study.

### 2.4. Psychometric Scales for Cognitive Functions

#### 2.4.1. Behavioral Inhibition/Activation Systems

The Behavioral Inhibition/Activation System Scale (BIS/BAS) assessed behavioral activation and inhibition [27]. The scale comprises 20 items, using a 4-point Likert scale ranging from 1 to 4 points. The behavioral inhibition system is composed of a single factor, while the behavioral activation system consists of three sub-factors representing a strong desire to engage in activities (‘Drive’), novelty-seeking behaviors with potential rewards (‘Fun-Seeking’), and positive responsiveness to rewards (‘Reward Responsiveness’). Higher scores on each factor indicate a higher propensity for the associated tendencies. The internal consistency (Cronbach’s α) in the Korean validation study was reported as 0.78 [28], while it was reported as 0.87 in this study.

#### 2.4.2. Impulsivity

The Barratt Impulsiveness Scale-11 (BIS-11) was used to quantify impulsivity [29]. The scale consists of 30 items, using a 4-point Likert scale ranging from 1 to 4 points. Higher total scores on the scale indicate higher levels of impulsivity. The internal consistency (Cronbach’s α) in the Korean validation study was reported as 0.80 [30], while it was reported as 0.87 in this study.

#### 2.4.3. Self-Control

The Brief Self-Control Scale (BSCS) measures the degree of self-control [31]. The scale comprises 11 items, using a 5-point Likert scale ranging from 1 to 5 points. Higher scores on the scale indicate higher levels of self-control ability. The internal consistency (Cronbach’s α) in the Korean validation study was reported as 0.81 [32], while it was reported as 0.89 in this study.

#### 2.4.4. Emotion Regulation

The Emotion Regulation Questionnaire (ERQ) was developed to examine emotion regulation [33]. The scale consists of 10 items, using a 7-point Likert scale ranging from 1 to 7 points. The ERQ comprises two factors—‘Reappraisal’, which reflects the cognitive reinterpretation of emotions in response to antecedent events, and ‘Suppression’, which reflects the tendency to inhibit the expression of one’s feelings. Higher scores on each factor indicate a higher propensity for the associated strategy. The reliability (Cronbach’s α) reported in the Korean validation study was 0.85 for reappraisal and 0.83 for suppression [34]. The reliability was 0.83 for reappraisal and 0.80 for suppression in this study.

### 2.5. Clinical Scales for Mental Health

#### 2.5.1. Depression

The Patient Health Questionnaire-9 (PHQ-9) was used to quantify depression symptoms [35]. The scale comprises nine items, with responses on a 4-point Likert scale ranging from 0 to 3 points. Total scores ranging from 0 to 4 indicate ‘no depression,’ scores between 5 and 9 indicate ‘mild depression,’ scores between 10 and 19 indicate ‘moderate depression,’ and scores of 20 or higher are classified as ‘severe depression’. The internal consistency (Cronbach’s α) in the Korean validation study was reported as 0.81 [36], while it was reported as 0.88 in this study.

#### 2.5.2. Stress

The Perceived Stress Scale (PSS) was used to assess the degree of perceived stress [37]. The scale consists of 10 items, using a 5-point Likert scale ranging from 0 to 4 points. Higher scores on the scale indicate a higher level of perceived stress. In the Korean validation study, the reliability (Cronbach’s α) was reported as 0.74 for positive perception and 0.77 for negative perception [38]. The reliability was 0.82 for positive perception and 0.90 for negative perception in this study.

#### 2.5.3. Anxiety

The State/Trait Anxiety Inventory-X (STAI-X) quantified the level of anxiety in each individual [39]. The inventory comprises 40 items on a 4-point Likert scale, with 20 items each for measuring state and trait anxiety. For state anxiety, total scores of 51 or below indicate ‘normal’, scores between 52 and 56 indicate ‘mild’, scores between 57 and 61 indicate ‘moderate’, and scores of 62 or higher suggest ‘severe’. For trait anxiety, total scores of 53 or below indicate ‘normal’, scores between 54 and 58 indicate ‘mild’, scores between 59 and 63 indicate ‘moderate’, and scores of 64 or higher suggest ‘severe’. The reliability (Cronbach’s α) reported in the Korean validation study was 0.87 for state anxiety and 0.86 for trait anxiety [40]. The reliability was 0.96 for state anxiety and 0.93 for trait anxiety in this study.

### 2.6. Statistical Analysis

Descriptive statistics were calculated to compare the general characteristics of the groups. Initially, the normality of demographic variables and clinical scale scores was assessed using the Shapiro–Wilk test, which indicated that the assumption of normality was violated for most variables.

Accordingly, group differences in demographic variables (gender, age, and BMI), BES, and addiction scales were assessed using the chi-square test and the Kruskal–Wallis test. In cases where a significant group difference was identified, post hoc comparisons with Bonferroni correction were applied. Bonferroni correction was applied within each scale, not across scales.

Subsequently, due to violations in the normality assumption and the positively skewed distribution of most scale scores, a generalized linear model (GLM) analysis with a gamma distribution and log-link function was conducted to examine group differences in cognitive and mental health characteristics. Gender and body mass index (BMI) were included as covariates to control for their effects. We also assessed models with alternative covariate variables, as shown in Table S2. As the PHQ-9 scale included zero values, a constant of 0.5 was added to all PHQ-9 scores to allow analysis under the gamma distribution. No other clinical scale contained zero values. The GLM results are reported as exponentiated β coefficients and their 95% confidence intervals (CIs), representing the relative difference in expected scores between each pair of groups. Specifically, an exponentiated β > 1 indicates that the first group in the comparison has a higher expected score than the reference group, whereas a value <1 suggests a lower expected score. Pairwise comparisons among all groups were conducted using Bonferroni correction within each scale. All statistical analyses were two-tailed and a *p*-value of less than 0.05 was considered statistically significant. All analyses were performed using R version 4.3.1.

## 3. Results

### 3.1. Demographic Information in the Study Groups

Based on the BES and four addiction scales (AUDIT, FTND, C-VAT, and CPGI), participants were classified into three groups—(1) the health control (HC) group, (2) the binge eating only (BE-only) group, and (3) the binge eating with comorbid addiction (BE+AD) group (as shown in Table 1). The HC group consisted of 180 participants who did not meet any clinical criteria for binge eating or addiction. The BE-only group comprised 32 participants who met the clinical cut-off for binge eating only. The BE+AD group included 30 participants who satisfied the clinical cut-off values in both binge eating behavior and at least one of the addiction screening scales. Among the 30 subjects in the BE+AD groups, 19 had a video game addiction, 16 had an alcohol addiction, 9 had a gambling disorder, and 1 subject had a nicotine dependence (13 participants had multiple addictions, while 17 had a single type of addiction). The mean number of addictive behaviors in this group was 1.5 (range: 1–3; SD: 0.62). The remaining 70 subjects with only addictive behaviors were not included in the present analysis.

As indicated in Table 1, a significant difference was observed in the gender ratio across the HC, BE-only, and BE+AD groups (gender: χ^2^(2) = 9.522, *p* = 0.009), whereas no significant difference was found in age or body mass index (BMI) among the three groups (age: χ^2^(2) = 1.657, *p* = 0.437; BMI: χ^2^(2) = 5.847, *p* = 0.054). The effects of gender and BMI were controlled in the GLM for the remaining data analyses, as BMI approached the significance level. We also conducted supplementary analyses using alternative GLMs. Model 2 includes only gender as a covariate, while Model 3 includes gender, BMI, and age as covariates (see Table S2). Additionally, an alternative model based solely on data from female participants was analyzed, as shown in Table S3. All significant group differences observed in the original GLM (Model 1) were also found in the alternative models.

### 3.2. Binge Eating and Addictions

#### 3.2.1. Binge Eating Scale

The BES scores showed a significant increase in both binge-eating groups compared to the HC group, as shown in Table 1. The Kruskal–Wallis test and post hoc test revealed significant differences between the HC and two binge-eating groups (i.e., BE-only and BE+AD) (χ^2^(2) = 138.190, *p* < 0.001). The two binge-eating groups were not significantly different (*p* = 0.370).

#### 3.2.2. Addiction Scales

Descriptive statistics and the Kruskal–Wallis test results regarding addiction scales (alcohol use, nicotine dependence, gaming, and gambling) are presented in Table 1. Only the BE+AD group showed significant differences from both the HC and the BE-only groups on all addiction scales, except for the FTND. The BE-only group was not significantly different from the HC group in any of the addiction scales (all *p* > 0.740, post hoc Bonferroni test).

### 3.3. Cognitive Characteristics

Descriptive statistics and the results of the gamma GLM regarding cognitive characteristics (behavioral activation/inhibition, impulsivity, self-control, and emotion regulation) are presented in Table 2.

#### 3.3.1. Behavioral Inhibition/Activation System (BIS/BAS)

In the BIS/BAS, only “Inhibition” showed a significant group difference, while the other three subfactors of BAS (Drive, Fun-seeking, and Reward Responsiveness) did not exhibit significant differences after Bonferroni correction. BIS/BAS—Inhibition scores were significantly higher in both the BE-only (exp(β) = 1.13; 95% CI: 1.05–1.21; *p* = 0.006) and the BE+AD groups compared to the HC group (exp(β) = 1.13; 95% CI: 1.05–1.22; *p* = 0.003). There was no significant difference between the two binge-eating groups (*p* = 1.000).

#### 3.3.2. Impulsivity (BIS-11)

Average BIS-11 scores showed a stepwise increase across three groups (mean ± SD: 57.71 ± 9.83, 63.16 ± 10.44, and 74.17 ± 14.31 for the HC, BE-only, and BE+AD groups, respectively). The gamma GLM revealed significant differences in BIS-11 scores among all group pairs—BE-only vs. HC (exp(β) = 1.10; 95% CI: 1.02–1.17; *p* = 0.029), BE+AD vs. HC (exp(β) = 1.29; 95% CI: 1.20–1.38; *p* < 0.001), and BE+AD vs. BE-only (exp(β) = 1.17; 95% CI: 1.08–1.28; *p* = 0.001).

#### 3.3.3. Self-Control (BSCS)

Average BSCS scores demonstrated a stepwise decrease across three groups (mean ± SD: 37.63 ± 8.04, 33.31 ± 8.29, and 26.30 ± 7.62 for the HC, BE-only, and BE+AD groups, respectively). The gamma GLM revealed significant differences in BSCS scores among all group pairs—the BE-only group scored lower than the HC group (exp(β) = 0.89; 95% CI: 0.81–0.97; *p* = 0.028); the BE+AD group scored lower than the HC group (exp(β) = 0.70; 95% CI: 0.64–0.76; *p* < 0.001); and the BE+AD group also scored lower than the BE-only group (exp(β) = 0.79; 95% CI: 0.70–0.89; *p* < 0.001).

#### 3.3.4. Emotion Regulation (ERQ)

In the ERQ, a significant difference among groups was observed only for the reappraisal subscale—the HC group had significantly higher scores than the BE+AD group (exp(β) = 0.84; 95% CI: 0.77–0.92; *p* < 0.001). No significant differences were found between the BE-only and HC groups (*p* = 0.777) or between the BE+AD and BE-only groups (*p* = 0.094).

### 3.4. Mental Health Characteristics

The descriptive statistics and the results of the gamma GLM regarding mental health characteristics (depression, stress, and anxiety) are presented in Table 2.

#### 3.4.1. Depression (PHQ-9)

The gamma GLM revealed a significant difference in PHQ-9 scores, with the BE+AD group scoring significantly higher than the HC group (exp(β) = 2.42; 95% CI: 1.75–3.42; *p* < 0.001). No significant differences were found between the BE-only and HC groups (*p* = 0.095) or between the BE+AD and BE-only groups (*p* = 0.057).

#### 3.4.2. Stress (PSS)

The PSS scores were substantially higher in both binge-eating groups compared to the HC group. The BE-only group scored significantly higher than the HC group (exp(β) = 1.30; 95% CI: 1.12–1.52; *p* = 0.002), and the BE+AD group also scored significantly higher than the HC group (exp(β) = 1.45; 95% CI: 1.25–1.69; *p* < 0.001). No significant difference was observed between the BE-only and BE+AD groups (*p* = 0.886).

#### 3.4.3. State and Trait Anxiety (STAI-X)

Both state and trait anxiety scores showed significant group differences. For state anxiety, the BE-only group had significantly higher scores than the HC group (exp(β) = 1.23; 95% CI: 1.10–1.38; *p* = 0.001), and the BE+AD group also scored significantly higher than the HC group (exp(β) = 1.34; 95% CI: 1.20–1.50; *p* < 0.001). No significant difference was observed between the BE-only and BE+AD groups (*p* = 0.712). Similarly, for trait anxiety, the BE-only group scored significantly higher than the HC group (exp(β) = 1.20; 95% CI: 1.08–1.32; *p* = 0.001), and the BE+AD group also scored significantly higher than the HC group (exp(β) = 1.34; 95% CI: 1.21–1.47; *p* < 0.001), with no significant difference observed between the BE-only and BE+AD groups (*p* = 0.261).

## 4. Discussion

We observed significant group differences across the HC, BE-only, and BE+AD groups in the following psychometric scales: BIS/BAS—Inhibition, BIS-11, BSCS, ERQ—Reappraisal, PHQ, PSS, and STAI-X (both state and trait anxiety). First, both binge-eating groups exhibited similar increases in BIS/BAS—Inhibition within the motivational system, as well as heightened perceived stress and anxiety, compared to the HC group. This suggests that individuals in both binge-eating groups are more sensitive to potential punishment and negative outcomes (high BIS/BAS—Inhibition), making them more susceptible to elevated stress and anxiety levels [41,42]. In accordance with our findings, Rosenbaum and White suggested that anxiety sensitivity and anxiety might be involved in binge eating development [43]. They also observed that stress and anxiety were associated with binge eating, independent of the impact of depression [44]. Stress, trait anxiety, and anxiety sensitivity were also implicated in binge eating studies with adolescent girls [45] and medical students [46]. Distress was also found to be associated with negative urgency and several types of maladaptive behaviors, including binge eating, alcohol use, and drug use, in a previous study [47]. In our study, binge eating was attributed to group differences in these traits of stress, anxiety, and punishment sensitivity, but the comorbid addiction did not alter these traits. Of particular note, even the BE+AD group—who might be expected to show elevated reward sensitivity due to their co-occurring addictive behaviors—did not show higher BAS—Drive scores. This finding aligns with prior studies reporting mixed or weak associations between reward sensitivity and binge eating pathology [42,48]. For example, Wilson et al. [42] reported that binge eating severity was positively associated with BIS anxiety-related items but negatively correlated with the BAS—Drive (or Goal-Directed Persistence) subscale [41,42]. This may suggest that the presence of addiction in binge-eating individuals does not necessarily manifest as increased reward motivation, at least as measured by the BAS—Drive scale. Alternatively, it may reflect heterogeneity in the types of addictive behaviors included (e.g., substance use vs. behavioral addictions) or limitations in the sensitivity of the BAS—Drive subscale. Taken together, these findings suggest that heightened punishment sensitivity—rather than attenuated reward sensitivity—may play a more consistent role in the psychopathology of binge eating.

Second, there was a significant increase in BIS-11 (impulsivity) and a decrease in BSCS (self-control) stepwise across the HC vs. BE-only vs. BE+AD groups. These factors might be associated with more severe psychopathological alterations in binge eating with comorbid addiction. The observed pattern suggests that impaired inhibitory control may represent a shared vulnerability factor across various clinical conditions, including eating disorders and addictions [6,9,49]. This is consistent with findings that impulsivity and compulsivity features are shared between gambling disorder and bulimic spectrum eating disorders, suggesting common neuropsychological profiles [11]. Previous studies have consistently demonstrated that individuals with binge eating exhibit elevated impulsivity and reduced self-control [50,51], but our findings extend this work by identifying a potential additive effect when addiction is comorbid. The significantly higher impulsivity and lower self-control in the BE+AD group may reflect compromised prefrontal cortical function, as neuroimaging studies have shown that both binge eating and addictions are associated with alterations in prefrontal regions responsible for executive control [8,9]. Alternatively, these traits may reflect a shared vulnerability that predisposes individuals to both binge eating and addiction, rather than an additive effect. This interpretation is consistent with transdiagnostic models of impulsivity, which link inhibitory control deficits to multiple forms of dysregulated behavior. This progressive impairment pattern suggests that interventions targeting impulsivity and self-control [52,53] might be particularly crucial for individuals with comorbid conditions.

Third, we observed unique alterations in the BE+AD groups for PHQ (depression) and ERQ—Reappraisal. Only the BE+AD group showed significantly stronger depression symptoms and impaired cognitive reappraisal in emotion regulation when compared to the HC group. This implies that these individuals may have difficulty managing negative emotions, potentially contributing to both binge eating and addictive behaviors. This aligns with research showing that individuals with BED tend to use maladaptive emotion regulation strategies more frequently than healthy controls [54]. The lack of significant differences in depression and cognitive reappraisal between the BE-only and HC groups is noteworthy. It suggests that the combination of binge eating and addictions may have a synergistic effect on mood and emotion regulation, which is not present in binge eating alone. In accordance with this, Farstad and von Ranson reported that women with both at-risk binge eating and at-risk problem gambling tendencies exhibited greater emotion dysregulation compared to those with either condition alone [16]. This finding underscores the importance of considering comorbid conditions when assessing and treating individuals with binge eating behaviors. For example, the co-occurrence of mood disorders was found to be more frequent in BED patients with substance use disorders in a previous study [14]. In another study, difficulty in emotion regulation was commonly observed in both eating disorders and gambling disorders, and was associated with poor treatment outcomes in these patients [55].

Notably, the prevalence of binge eating was relatively higher in the present study (62 out of 312 subjects; 19.9%) than the previously reported prevalence rate of binge eating in the general population (e.g., 13.0% in Australia [3] and 6.2% in Brazil [4]). As recruitment was conducted online, it may have drawn increased engagement from individuals with concerns about eating or addictive behaviors, potentially introducing bias in age and gender distributions. Moreover, the recruitment advertisement was explicitly titled “Online Behavioral Survey Research Participants Wanted for Addiction Proneness,” listing various domains of addictive behaviors, including food, alcohol, nicotine, gambling, and gaming (see Supplementary Information for full text). This wording may have further increased the likelihood of attracting individuals already concerned with these behaviors, potentially contributing to the elevated prevalence of binge eating observed in our sample. Nonetheless, participants were subsequently grouped using validated clinical screening cut-offs, which were independent of the recruitment context, and this design may have helped reduce the potential impact of self-selection bias on group comparisons.

In the present study, most participants were young females (as described in the Methods and Table 1) who had an elevated risk of binge eating and eating disorders. While our sample may not fully represent the general Korean population, its demographic profile aligns with known risk patterns of eating disorders, particularly the higher prevalence in females and younger adults [56,57]. Of note, the observed prevalence of binge eating symptoms (19.9%) is notably higher than what has been reported in Korean epidemiological studies [56,57], which may further limit the generalizability of the findings to the broader population. In the absence of large-scale epidemiological studies on BED in Korea, studies focusing on such at-risk groups may serve as a preliminary step toward characterizing the psychological features of binge eating in specific populations.

Furthermore, the present study screened individuals with binge eating problems using the self-reported screening tool, i.e., BES, which might be less accurate than clinical interviews. Although this tool has demonstrated a high sensitivity and specificity in other populations (e.g., 81.8% and 97.8% in Portuguese females) [58], it has not been fully validated in Korean samples, raising the possibility that subclinical cases may have been included. Nevertheless, all participants were classified using the same standardized criteria, and key covariates (e.g., gender and BMI) were statistically controlled in the analyses, supporting the internal validity of group comparisons between the BE-only and BE+AD groups.

In contrast to previous studies, the present study assessed frequent substance addictions (alcohol and nicotine) and behavioral addictions (gambling and video games) together to identify the comorbid addiction group. Among 30 BE+AD subjects, 17 had substance addiction (16 for alcohol and 1 for nicotine), but the remaining 13 had only behavioral addictions. These binge-eating individuals with co-occurring behavioral addictions might have been ignored so far in previous studies of the comorbidity of addiction in the binge-eating population. While it might be worthwhile to contrast the effects of substance addictions vs. behavioral addictions in the binge-eating population, we abandoned it due to the limited sample size and the heterogeneity of the BE+AD group in the present study (e.g., some participants also exhibited both types of addiction). However, the present study design is readily scalable to a larger population when combined with online survey tools. Further studies with a larger sample size could provide more concrete evidence through detailed subgroup comparisons (e.g., the effect of the types and numbers of comorbid addictions).

There are a few other limitations in the present study. First, screening of the addictive behaviors was based on self-reported clinical questionnaires. Thus, it might be less accurate and inflate the prevalence of each addiction. However, the self-report-based study has its advantages in scalability, as noted above. Alternatively, future studies could incorporate clinical interviews conducted by trained professionals to validate self-reported diagnoses of binge eating and addictions. Additionally, combining self-report measures with objective behavioral tasks (e.g., inhibitory control tasks) or neuroimaging techniques could provide a more comprehensive understanding of the underlying mechanisms. Second, the cross-sectional nature of this study limits the ability to infer causal relationships between binge eating, comorbid addiction, and cognitive or emotional traits. For example, it remains unclear whether increased impulsivity and reduced self-control are precursors to binge eating and addictions or consequences of these behaviors. Longitudinal studies tracking participants over time could help establish causal pathways and identify potential mediators or moderators of these relationships. Third, although the sample size was sufficient to detect significant differences between groups, the BE+AD group consisted of a relatively small number of participants with varying types of addictions (e.g., alcohol, nicotine, gambling, and video gaming). This heterogeneity may have introduced variability that limited the ability to detect specific effects related to particular types of addiction. For example, only one participant in the BE+AD group had nicotine dependence, making it impossible to draw any reliable conclusions regarding its specific effects. Future studies with larger samples are needed to assess the role of nicotine addiction more systematically. More generally, future research should aim for larger sample sizes with a more balanced representation across different types of substance and behavioral addictions. Stratifying participants by addiction type could help clarify whether certain addictions interact differently with binge eating.

## 5. Conclusions

Our study is a systematic evaluation of a range of cognitive functions and mental health characteristics that are commonly implicated in binge eating and addictions. Increased impulsivity and reduced self-control are major predisposing factors for binge eating and addictive behaviors, whereas impaired emotion regulation and more severe depressive symptoms are particularly pronounced in comorbid conditions. While Bonferroni correction was applied within outcome domains, the study had an exploratory design and did not correct for all comparisons. Thus, the findings should be considered preliminary and require validation in future research with larger samples. Rather than isolated traits, the observed cognitive profiles suggest transdiagnostic mechanisms that may underlie compulsive behaviors across binge eating and addiction. These findings highlight specific intervention targets—such as impulsivity, diminished self-control, and emotion regulation deficits—that are clinically modifiable through cognitive–behavioral therapy approaches. By identifying these shared cognitive vulnerabilities, the study offers practical guidance for developing more effective, mechanism-based treatments for individuals struggling with binge eating and related addictive behaviors.

The present study served as an initial step in characterizing cognitive and mental health profiles among binge-eating individuals with and without comorbid addictions. Future research should aim to expand on these findings in several ways. First, studies with larger and more balanced samples are needed to enable more detailed subgroup comparisons and robust statistical modeling. In particular, mediation and moderation analyses could clarify the potential mechanisms through which cognitive traits (e.g., impulsivity and emotion regulation) influence the relationship between binge eating and comorbid addictive behaviors. Such analyses would benefit from longitudinal data and theoretically driven hypotheses to ensure causal interpretability. Second, incorporating structured clinical interviews alongside self-report measures would enhance diagnostic validity. Finally, complementing psychometric data with behavioral or neurobiological measures (e.g., inhibitory control tasks and neuroimaging) may provide a more comprehensive understanding of the shared and distinct mechanisms underlying binge eating and addictions. Taken together, these future directions will build upon the current findings and contribute to a more refined and mechanistic understanding of binge eating and its comorbidity with addiction, ultimately informing more targeted and effective interventions.

## Figures and Tables

**Table 1:** The demographic information and the addiction profiles of the study groups.

Groups	HC (*n* = 180)	BE-Only (*n* = 32)	BE+AD (*n* = 30)	$\chi^{2}$	*p*-Value
Gender (Female)	113 (62.92%)	29 (90.63%)	20 (66.67%)	9.522	**0.009**
Age (year)	28.60 (9.76)	30.03 (8.51)	28.43 (9.02)	1.657	0.437
BMI (kg/m^2^)	22.05 (3.27)	23.70 (3.96)	22.95 (3.36)	5.847	0.054
BES	7.37 (4.80)	24.75 (6.88)	26.73 (6.37)	138.190	**<0.001 ^ab^**
AUDIT	4.85 (3.52)	4.78 (4.11)	11.63 (9.39)	13.633	**0.001 ^bc^**
FTND	0.17 (0.74)	0.19 (0.78)	1.00 (1.91)	14.602	**<0.001 ^b^**
C-VAT	0.82 (1.36)	0.69 (1.53)	5.80 (4.01)	46.093	**<0.001 ^bc^**
CPGI	0.23 (0.81)	0.38 (0.94)	3.90 (5.18)	28.431	**<0.001 ^bc^**

Note: mean (SD) except for the gender. BMI: body mass index; BES: Binge Eating Scale; AUDIT: Alcohol Use Disorders Identification Test; FTND: Fagerstrom Test for Nicotine Dependence; C-VAT: Clinical Video Game Addiction Test 2.0; CPGI: Canadian Problem Gambling Index. Significant *p* values appear in bold. **a**: *p* < 0.05 for HC vs. BE-only; **b**: *p* < 0.05 for HC vs. BE+AD; **c**: *p* < 0.05 for BE-only vs. BE+AD.

**Table 2:** The gamma GLM results of psychometric variables.

	Mean (SD)			Exp(β) (Bonferroni Corrected *p*-Values)		
	HC (*n* = 180)	BE-Only (*n* = 32)	BE+AD (*n* = 30)	BE-Only vs. HC	BE+AD vs. HC	BE+AD vs. BE-Only
BIS/BAS: Inhibition	19.68 (3.91)	22.53 (3.08)	22.30 (3.97)	**1.13** **(0.006)**	**1.13** **(0.003)**	1.00 (>0.99)
BIS/BAS: Drive	11.02 (1.96)	11.81 (2.58)	10.57 (2.80)	1.08 (0.164)	0.96 (0.966)	0.89 (0.071)
BIS/BAS: Fun	10.17 (2.35)	11.06 (2.37)	11.03 (2.99)	1.10 (0.164)	1.09 (0.225)	0.99 (>0.99)
BIS/BAS: Reward	14.86 (2.51)	16.06 (2.95)	15.80 (2.40)	1.08 (0.084)	1.06 (0.201)	0.99 (>0.99)
BIS-11	57.71 (9.83)	63.16 (10.44)	74.17 (14.31)	**1.10** **(0.029)**	**1.29** **(<0.001)**	**1.17** **(0.001)**
BSCS	37.63 (8.04)	33.31 (8.29)	26.30 (7.62)	**0.89** **(0.028)**	**0.70** **(<0.001)**	**0.79** **(<0.001)**
ERQ: Reappraisal	29.00 (6.06)	27.97 (6.36)	24.37 (7.24)	0.95 (0.777)	**0.84** **(<0.001)**	0.88 (0.094)
ERQ: Suppression	16.43 (5.01)	15.44 (5.99)	17.53 (5.27)	0.96 (>0.99)	1.07 (0.813)	1.11 (0.550)
PHQ-9	4.22 (4.16)	6.16 (5.20)	10.73 (6.88)	1.45 (0.095)	**2.42** **(<0.001)**	1.67 (0.057)
PSS	16.58 (6.75)	21.69 (7.08)	23.97 (6.21)	**1.30** **(0.002)**	**1.45** **(<0.001)**	1.11 (0.886)
STAI-X: State	41.45 (12.18)	50.31 (13.61)	55.30 (12.58)	**1.23** **(0.001)**	**1.34** **(<0.001)**	1.09 (0.712)
STAI-X: Trait	42.53 (11.23)	50.41 (10.23)	56.50 (10.60)	**1.20** **(0.001)**	**1.34** **(<0.001)**	1.12 (0.261)

Note: BIS/BAS: Behavioral Inhibition/Activation System Scale. BIS-11: Barratt Impulsiveness Scale-11. BSCS: Brief Self-Control Scale. ERQ: Emotion Regulation Questionnaire. PHQ-9: Patient Health Questionnaire-9. PSS: Perceived Stress Scale. STAI-X: State/Trait Anxiety Inventory-X. Significant *p* values appear in bold.

## Data Availability

The raw data supporting the conclusions of this article will be made available by the authors on request.

## Supplementary Materials

The following supporting information can be downloaded at: https://www.mdpi.com/article/10.3390/healthcare13131524/s1, Online advertisement for recruiting participants (English translation); Table S1: Distribution of participants meeting criteria for each type of addiction; Table S2: Supplementary descriptive statistics of psychometric variables; Table S3: Supplementary analyses of gamma GLMs with alternative covariates; Table S4: Supplementary analyses of gamma GLMs for gender effect; Figure S1: Boxplots for comparing demographic variables and clinical scales across 3 study groups.

## Abbreviations

The following abbreviations are used in this manuscript:

AUD	Alcohol use disorder
AUDIT	Alcohol Use Disorders Identification Test
BED	Binge eating disorder
BES	Binge Eating Scale
BIS-11	Barratt Impulsiveness Scale-11
BMI	Body mass index
BN	Bulimia nervosa
BSCS	Brief Self-Control Scale
CPGI	Canadian Problem Gambling Index
CI	Confidence Interval
C-VAT 2.0	Clinical Video Game Addiction Test 2.0
ERQ	Emotion Regulation Questionnaire
FTND	Fagerstrom Test for Nicotine Dependence
GLM	Generalized linear model
PHQ-9	Patient Health Questionnaire-9
PSS	Perceived Stress Scale
SD	Standard deviation
STAI-X	State/Trait Anxiety Inventory-X

## References

[1] American Psychiatric Association, DSMTF, DS American Psychiatric Association (2013). Diagnostic and Statistical Manual of Mental Disorders: DSM-5.

[2] Stunkard A.J. (1959). Eating patterns and obesity. Psychiatr. Q..

[3] Mitchison D., Touyz S., González-Chica D.A., Stocks N., Hay P. (2017). How abnormal is binge eating? 18-year time trends in population prevalence and burden. Acta Psychiatr. Scand..

[4] Appolinario J.C., Sichieri R., Lopes C.S., Moraes C.E., da Veiga G.V., Freitas S., Nunes M.A., Wang Y.-P., Hay P. (2022). Correlates and impact of DSM-5 binge eating disorder, bulimia nervosa and recurrent binge eating: A representative population survey in a middle-income country. Soc. Psychiatry Psychiatr. Epidemiol..

[5] Kessler R.C., Berglund P.A., Chiu W.T., Deitz A.C., Hudson J.I., Shahly V., Aguilar-Gaxiola S., Alonso J., Angermeyer M.C., Benjet C. (2013). The prevalence and correlates of binge eating disorder in the World Health Organization World Mental Health Surveys. Biol. Psychiatry.

[6] Michaud A., Vainik U., Garcia-Garcia I., Dagher A. (2017). Overlapping neural endophenotypes in addiction and obesity. Front. Endocrinol..

[7] Yu Y., Miller R., Groth S.W. (2022). A literature review of dopamine in binge eating. J. Eat. Disord..

[8] Pasquale E.K., Boyar A.M., Boutelle K.N. (2024). Reward and Inhibitory Control as Mechanisms and Treatment Targets for Binge Eating Disorder. Curr. Psychiatry Rep..

[9] Schulte E.M., Grilo C.M., Gearhardt A.N. (2016). Shared and unique mechanisms underlying binge eating disorder and addictive disorders. Clin. Psychol. Rev..

[10] Mestre-Bach G., Potenza M.N. (2023). Potential biological markers and treatment implications for binge eating disorder and behavioral addictions. Nutrients.

[11] Lozano-Madrid M., Granero R., Lucas I., Sánchez I., Sánchez-González J., Gómez-Peña M., Moragas L., Mallorquí-Bagué N., Tapia J., Jiménez-Murcia S. (2023). Impulsivity and compulsivity in gambling disorder and bulimic spectrum eating disorders: Analysis of neuropsychological profiles and sex differences. Eur. Psychiatry.

[12] Kowalewska E., Bzowska M., Engel J., Lew-Starowicz M. (2024). Comorbidity of binge eating disorder and other psychiatric disorders: A systematic review. BMC Psychiatry.

[13] Peterson C.B., Miller K.B., Crow S.J., Thuras P., Mitchell J.E. (2005). Subtypes of binge eating disorder based on psychiatric history. Int. J. Eat. Disord..

[14] Becker D.F., Grilo C.M. (2015). Comorbidity of mood and substance use disorders in patients with binge-eating disorder: Associations with personality disorder and eating disorder pathology. J. Psychosom. Res..

[15] Devoe D.J., Anderson A., Bahji A., Singh M., Patten S.B., Soumbasis A., Ramirez Pineda A., Flanagan J., Richardson C., Lange T. (2022). The prevalence of impulse control disorders and behavioral addictions in eating disorders: A systematic review and meta-analysis. Front. Psychiatry.

[16] Farstad S.M., von Ranson K.M. (2021). Binge eating and problem gambling are prospectively associated with common and distinct deficits in emotion regulation among community women. Can. J. Behav. Sci./Rev. Can. Des Sci. Du Comport..

[17] Gormally J., Black S., Daston S., Rardin D. (1982). The assessment of binge eating severity among obese persons. Addict. Behav..

[18] Lee S., Hyun M. (2001). The effects of obesity, body image satisfaction, and binge eating on depression in middle school girls. Korean J. Health Psychol..

[19] Babor T.F., Higgins-Biddle J.C., Saunders J.B., Monteiro M.G., World Health Organization (2001). AUDIT: The Alcohol Use Disorders Identification Test: Guidelines for Use in Primary Care.

[20] Lee B. (2000). Development of Korean version of alcohol use disorders identification test (AUDIT-K): Its reliability and validity. J. Korean Acad. Addict. Psychiatry.

[21] Heatherton T.F., Kozlowski L.T., Frecker R.C., Fagerstrom K.O. (1991). The Fagerström test for nicotine dependence: A revision of the Fagerstrom Tolerance Questionnaire. Br. J. Addict..

[22] Ahn H., Lee H., Jung D., Lee S., Kim S., Kang J. (2002). The Reliability and Validity of Korean Version of Questionnaire for Nicotine Dependence. J. Korean Acad. Fam. Med..

[23] Van Rooij A.J., Schoenmakers T.M., Van de Mheen D. (2017). Clinical validation of the C-VAT 2.0 assessment tool for gaming disorder: A sensitivity analysis of the proposed DSM-5 criteria and the clinical characteristics of young patients with ‘video game addiction’. Addict. Behav..

[24] Jang S., Jun D., Lee M., Shin S. (2020). A validation of the Korean version of the clinical video game addiction test 2.0. J. Rehabil. Psychol..

[25] Ferris J.A., Wynne H.J. (2001). The Canadian Problem Gambling Index.

[26] Kim A., Cha J., Kwon S., Lee S. (2011). Construction and validation of Korean version of CPGI. Korean J. Psychol. Gen..

[27] Carver C.S., White T.L. (1994). Behavioral inhibition, behavioral activation, and affective responses to impending reward and punishment: The BIS/BAS scales. J. Personal. Soc. Psychol..

[28] Kim K., Kim W. (2001). Korean—BAS/BIS Scale. Korean J. Health Psychol..

[29] Patton J.H., Stanford M.S., Barratt E.S. (1995). Factor structure of the Barratt impulsiveness scale. J. Clin. Psychol..

[30] Lee S., Lee W., Park J., Kim S., Kim J., Shim J. (2012). The study on Reliability and Validity of Korean Version of the Barratt Impulsiveness Scale-11-Revised in nonclinical adult subjects. J. Korean Neuropsychiatr. Assoc..

[31] Tangney J.P., Boone A.L., Baumeister R.F. (2018). High self-control predicts good adjustment, less pathology, better grades, and interpersonal success. Self-Regulation and Self-Control.

[32] Hong H., Kim H., Kim J., Kim J. (2012). Validity and reliability validation of the Korean version of the Brief Self-Control Scale (BSCS). Korean J. Psychol. Gen..

[33] Gross J.J., John O.P. (2003). Individual differences in two emotion regulation processes: Implications for affect, relationships, and well-being. J. Personal. Soc. Psychol..

[34] Shon J. (2005). Individual Differences in Two Regulation Strategies: Cognitive Reappraiser vs. Emotion Suppressor. Master’s Thesis.

[35] Kroenke K., Spitzer R.L., Williams J.B. (2001). The PHQ-9: Validity of a brief depression severity measure. J. Gen. Intern. Med..

[36] Park S., Choi H., Choi J., Kim K., Hong J. (2010). Reliability and validity of the Korean version of the Patient Health Questionnaire-9 (PHQ-9). Anxiety Mood.

[37] Cohen S., Kamarck T., Mermelstein R. (1983). A global measure of perceived stress. J. Health Soc. Behav..

[38] Park J., Seo Y. (2010). Validation of the perceived stress scale (PSS) on samples of Korean university students. Korean J. Psychol. Gen..

[39] Spielberger C.D., Gorsuch R.L., Lushene R.E. (1970). Manual for the State-Trait Anxiety Inventory.

[40] Kim J., Sin D. (1978). The Relationship Between Trait Anxiety and Social Tendency-Based on Spielberger’s STAI. Master’s Thesis.

[41] Bijttebier P., Beck I., Claes L., Vandereycken W. (2009). Gray’s Reinforcement Sensitivity Theory as a framework for research on personality–psychopathology associations. Clin. Psychol. Rev..

[42] Wilson D.R., Loxton N.J., O’Donovan A. (2021). From BIS to binge: The role of negative affect in the pathway between personality and binge eating. Eat. Behav..

[43] Rosenbaum D.L., White K.S. (2013). The role of anxiety in binge eating behavior: A critical examination of theory and empirical literature. Health Psychol. Res..

[44] Rosenbaum D.L., White K.S. (2015). The relation of anxiety, depression, and stress to binge eating behavior. J. Health Psychol..

[45] Jung J., Kim K., Woo H., Shin D., Shin Y., Oh K., Shin E., Lim S. (2017). Binge eating is associated with trait anxiety in Korean adolescent girls: A cross sectional study. BMC Women’s Health.

[46] Torres M.F.B., Landeros O.G., Cosme J.A.G., Sandoval L.R.A. (2024). Binge eating disorder symptomatology and its association with depression, anxiety and stress: A cross-sectional study in medical students. Rev. Mex. De Trastor. Aliment..

[47] Borg D., Hall K., Youssef G.J., Sloan E., Graeme L., Moulding R. (2022). Examining the role of brooding, distress, and negative urgency in dysregulated behaviors: A cross-sectional study in treatment-seeking young people. J. Clin. Psychol..

[48] Oliva R., Budisavljević S., Castiello U., Begliomini C. (2021). Neuroanatomical correlates of binge-eating behavior: At the roots of unstoppable eating. Brain Sci..

[49] Meule A. (2013). Impulsivity and overeating: A closer look at the subscales of the Barratt Impulsiveness Scale. Front. Psychol..

[50] Annagur B.B., Orhan O., Ozer A., Yalcin N., Tamam L. (2015). The effects of depression and impulsivity on obesity and binge eating disorder. Klin. Psikofarmakol. Bülteni-Bull. Clin. Psychopharmacol..

[51] Ivezaj V., White M.A., Grilo C.M. (2016). Examining binge-eating disorder and food addiction in adults with overweight and obesity. Obesity.

[52] Javidan L., Meftahi A., Dabiri T., Solgi Z. (2023). The Effectiveness of Strengths-based Treatments for Impulsivity and Self-control in Female Adolescents With Binge-eating Disorders. Casp. J. Health Res..

[53] Schag K., Rennhak S.K., Leehr E.J., Skoda E.-M., Becker S., Bethge W., Martus P., Zipfel S., Giel K.E. (2019). IMPULS: Impulsivity-focused group intervention to reduce binge eating episodes in patients with binge eating disorder–a randomised controlled trial. Psychother. Psychosom..

[54] Dingemans A., Danner U., Parks M. (2017). Emotion regulation in binge eating disorder: A review. Nutrients.

[55] Vintró-Alcaraz C., Munguía L., Granero R., Gaspar-Pérez A., Solé-Morata N., Sánchez I., Sánchez-González J., Menchón J.M., Jiménez-Murcia S., Fernández-Aranda F. (2022). Emotion regulation as a transdiagnostic factor in eating disorders and gambling disorder: Treatment outcome implications. J. Behav. Addict..

[56] Lee S., Hong M., Park S., Kang W., Oh I. (2021). Economic burden of eating disorders in South Korea. J. Eat. Disord..

[57] Cho M., Sung S., Shin S., Kim J., Cheon S., Kim M. (2012). The Epidemiological Survey of Mental Disorders in Korea 2011.

[58] Duarte C., Pinto-Gouveia J., Ferreira C. (2015). Expanding binge eating assessment: Validity and screening value of the Binge Eating Scale in women from the general population. Eat. Behav..

