# Supplementary material for: Cognitive and Mental Health Profiles of Binge-Eating Adults with and Without Comorbid Addictive Behaviors
Source: Healthcare (Basel). 2025 Jun 26;13(13):1524. doi: 10.3390/healthcare13131524 (PMC12249126; doi:10.3390/healthcare13131524)
Supplement: Supplementary file 1 [file healthcare-13-01524-s001.zip › healthcare-3619487-supplementary.pdf]

Cognitive and Mental Health Profiles of Binge-Eating Adults with and without Comorbid Addictive Behaviors

Jake Jeong [1,2], Jungwon Jang [1,2], Giho Jeon [1,2] and Kwangyeol Baek [1,2,3*]

Supplementary Methods

Online advertisement for recruiting participants (English translation)

Title: Research Participants Wanted for Online Behavioral Survey on Addiction Proneness

1. Research Purpose

We are conducting online psychological and behavioral surveys to analyze individual addiction-related factors.

- Alcohol Addiction
- Nicotine Addiction
- Caffeine Addiction
- Food Addiction
- Gambling Addiction
- Gaming Addiction
- Internet Addiction
- Shopping Addiction

2. Eligibility for Participation

Voluntary participants aged 19 to 59 who can participate in online surveys using a PC or smartphone (excluding those with difficulties in Korean communication, currently undergoing treatment for mental illness).

3. How to Participate in the Research

Time Required: Approximately 1 to 1.5 hours for survey completion and behavioral task participation.

What You Will Receive: Psychological test results and feedback (depression, anxiety, stress, etc.).

Participation Fee: A small honorarium will be provided upon completion of the study.

Supplementary Table S1. Distribution of participants meeting criteria for each type of addiction. This table presents the types of addictive behaviors identified among participants with both binge-eating and addictive behaviors (i.e., BE+AD group) in the study. The category of multiple addictions includes participants who met the criteria for two or more types of addictive behaviors.

**BE+AD group (n=30)**	**Addictive behaviors (alcohol, video games, nicotine, gambling)**	**No. of subjects**
**Multiple Addiction (n=13)**	alcohol, video games, & gambling	2
	Alcohol & video games	5
	Alcohol & gambling	3
	Nicotine & video games	1
	Video games & gambling	2
**Single Addiction (n=17)**	alcohol	6
	video games	9
	nicotine	0
	gambling	2

Supplementary Table S2. Supplementary descriptive statistics of psychometric variables.
This table presents the median and interquartile range (IQR) of psychometric variables for each group.

		Median (IQR)	
	**HC (n = 180)**	**BE-only (n = 32)**	**BE+AD (n = 30)**
BIS/BAS: Inhibition	20.0 (5.00)	21.0 (5.00)	22.0 (7.00)
BIS/BAS: Drive	11.0 (2.00)	11.5 (4.00)	11.0 (3.75)
BIS/BAS: Fun	10.0 (3.00)	11.0 (2.50)	12.0 (4.00)
BIS/BAS: Reward	15.0 (2.00)	16.0 (4.25)	16.0 (2.00)
BIS-11	57.0 (13.00)	61.5 (13.50)	75.0 (14.75)
BSCS	37.5 (12.00)	34.5 (11.00)	25.5 (8.50)
ERQ: Reappraisal	29.0 (7.00)	26.0 (8.50)	24.0 (6.50)
ERQ: Suppression	17.0 (7.00)	15.0 (8.50)	17.5 (8.75)
PHQ-9	3.0 (5.00)	4.5 (4.25)	9.0 (11.25)
PSS	16.0 (8.00)	21.0 (6.25)	22.5 (7.75)
STAI-X: State	39.0 (15.25)	48.5 (19.50)	53.0 (15.50)
STAI-X: Trait	41.0 (16.25)	47.5 (14.25)	55.5 (15.00)

Supplementary Table S3. Supplementary analyses of gamma GLMs with alternative covariates.
This table presents the exponentiated coefficients and Bonferroni-corrected p-values from additional gamma GLM analyses. Model 1 is the original model described in the manuscript. Model 2 excludes BMI from the covariates, while Model 3 includes age, which was not originally adjusted for. All significant pairs of comparison in Model 1 remain to be significant in Model 2 and 3.

Model 1: Scales = Group + gender + BMI
Model 2: Scales = Group + gender
Model 3: Scales = Group + gender + BMI + age

	**Exp(β) (Bonferroni corrected p-values)**								
	**Model 1**			**Model 2**			**Model 3**		
	BE-only vs. HC	BE+AD vs. HC	BE+AD vs. BE-only	BE-only vs. HC	BE+AD vs. HC	BE+AD vs. BE-only	BE-only vs. HC	BE+AD vs. HC	BE+AD vs. BE-only
BIS/BAS: Inhibition	**1.13 (0.006)**	**1.13 (0.003)**	1.00 (>0.99)	**1.12 (0.006)**	**1.13 (0.004)**	1.01 (>0.99)	**1.13 (0.006)**	**1.13 (0.003)**	1.00 (>0.99)
BIS/BAS: Drive	1.08 (0.164)	0.96 (0.966)	0.89 (0.071)	1.07 (0.266)	0.96 (0.819)	0.90 (0.097)	1.08 (0.174)	0.96 (0.913)	0.89 (0.070)
BIS/BAS: Fun	1.10 (0.164)	1.09 (0.225)	0.99 (>0.99)	1.09 (0.221)	1.08 (0.249)	1.00 (>0.99)	1.09 (0.168)	1.09 (0.226)	0.99 (>0.99)
BIS/BAS: Reward	1.08 (0.084)	1.06 (0.201)	0.99 (>0.99)	1.07 (0.102)	1.06 (0.228)	0.99 (>0.99)	1.08 (0.072)	1.06 (0.187)	0.99 (>0.99)
BIS-11	**1.10 (0.029)**	**1.29 (<0.001)**	**1.17 (0.001)**	**1.09 (0.033)**	**1.28 (<0.001)**	**1.18 (<0.001)**	**1.10 (0.028)**	**1.29 (<0.001)**	**1.17 (0.001)**
BSCS	**0.89 (0.028)**	**0.70 (<0.001)**	**0.79 (<0.001)**	**0.89 (0.027)**	**0.70 (<0.001)**	**0.79 (<0.001)**	**0.89 (0.031)**	**0.70 (<0.001)**	**0.79 (<0.001)**
ERQ: Reappraisal	0.95 (0.777)	**0.84 (<0.001)**	0.88 (0.094)	0.95 (0.854)	**0.84 (<0.001)**	0.88 (0.080)	0.95 (0.765)	**0.84 (<0.001)**	0.88 (0.095)
ERQ: Suppression	0.96 (>0.99)	1.07 (0.813)	1.11 (0.550)	0.97 (>0.99)	1.07 (0.753)	1.11 (0.574)	0.96 (>0.99)	1.07 (0.779)	1.12 (0.536)
PHQ-9	1.45 (0.095)	**2.42 (<0.001)**	1.67 (0.057)	1.41 (0.114)	**2.38 (<0.001)**	**1.69 (0.049)**	1.42 (0.118)	**2.43 (<0.001)**	**1.70 (0.046)**
PSS	**1.30 (0.002)**	**1.45 (<0.001)**	1.11 (0.886)	**1.30 (0.002)**	**1.44 (<0.001)**	1.11 (0.836)	**1.30 (0.002)**	**1.45 (<0.001)**	1.11 (0.880)
STAI-X: State	**1.23 (0.001)**	**1.34 (<0.001)**	1.09 (0.712)	**1.21 (0.002)**	**1.33 (<0.001)**	1.10 (0.591)	**1.23 (0.001)**	**1.35 (<0.001)**	1.09 (0.638)
STAI-X: Trait	**1.20 (0.001)**	**1.34 (<0.001)**	1.12 (0.261)	**1.18 (0.002)**	**1.33 (<0.001)**	1.12 (0.202)	**1.20 (0.001)**	**1.34 (<0.001)**	1.12 (0.261)

Note: Significant p values appear in bold.

Supplementary Table S4. Supplementary analyses of Gamma GLMs for gender effect. This table presents the exponentiated coefficients and Bonferroni-corrected p-values from an additional gamma GLM analysis (Model 4), which includes only female participants. Model 1 is the original model described in the manuscript. All significant pairs of comparison in Model 1 remain to be significant in Model 4.

Model 1: Scales = Group + gender + BMI
Model 4: Scales = Group + BMI (female participants only)

	Exp(β) (Bonferroni corrected p-values)					
	**Model 1**			**Model 4**		
	BE-only vs. HC	BE+AD vs. HC	BE+AD vs. BE-only	BE-only vs. HC	BE+AD vs. HC	BE+AD vs. BE-only
BIS/BAS: Inhibition	**1.13 (0.006)**	**1.13 (0.003)**	1.00 (>0.99)	**1.13 (0.006)**	**1.15 (0.004)**	1.02 (>0.99)
BIS/BAS: Drive	1.08 (0.164)	0.96 (0.966)	0.89 (0.071)	1.05 (0.666)	0.94 (0.608)	0.89 (0.153)
BIS/BAS: Fun	1.10 (0.164)	1.09 (0.225)	0.99 (>0.99)	1.09 (0.249)	**1.15 (0.031)**	1.06 (>0.99)
BIS/BAS: Reward	1.08 (0.084)	1.06 (0.201)	0.99 (>0.99)	1.07 (0.153)	**1.11 (0.033)**	1.04 (>0.99)
BIS-11	**1.10 (0.029)**	**1.29 (<0.001)**	**1.17 (0.001)**	**1.11 (0.016)**	**1.30 (<0.001)**	**1.17 (0.008)**
BSCS	**0.89 (0.028)**	**0.70 (<0.001)**	**0.79 (<0.001)**	**0.85 (0.006)**	**0.67 (<0.001)**	**0.78 (0.001)**
ERQ: Reappraisal	0.95 (0.777)	**0.84 (<0.001)**	0.88 (0.094)	0.96 (>0.99)	**0.82 (0.001)**	**0.85 (0.040)**
ERQ: Suppression	0.96 (>0.99)	1.07 (0.813)	1.11 (0.550)	0.94 (>0.99)	1.10 (0.748)	1.16 (0.331)
PHQ-9	1.45 (0.095)	**2.42 (<0.001)**	1.67 (0.057)	1.51 (0.060)	**2.52 (<0.001)**	1.67 (0.104)
PSS	**1.30 (0.002)**	**1.45 (<0.001)**	1.11 (0.886)	**1.31 (0.004)**	**1.47 (<0.001)**	1.13 (0.841)
STAI-X: State	**1.23 (0.001)**	**1.34 (<0.001)**	1.09 (0.712)	**1.23 (0.002)**	**1.33 (<0.001)**	1.08 (>0.99)
STAI-X: Trait	**1.20 (0.001)**	**1.34 (<0.001)**	1.12 (0.261)	**1.21 (0.001)**	**1.34 (<0.001)**	1.11 (0.489)

Note: Significant p values appear in bold.

Supplementary Figure S1. Boxplots for comparing demographic variables and clinical scales across 3 study groups, i.e., Healthy Controls (HC), Binge-eating only (BE-only), and Binge-eating with addictive behaviors (BE+AD). The cut-off scores used in this study are indicated by red dashed lines. Each dot represents an individual participant. For the FTND, C-VAT 2.0, and CPGI scales, data points clustered at zero were not displayed for visual clarity.

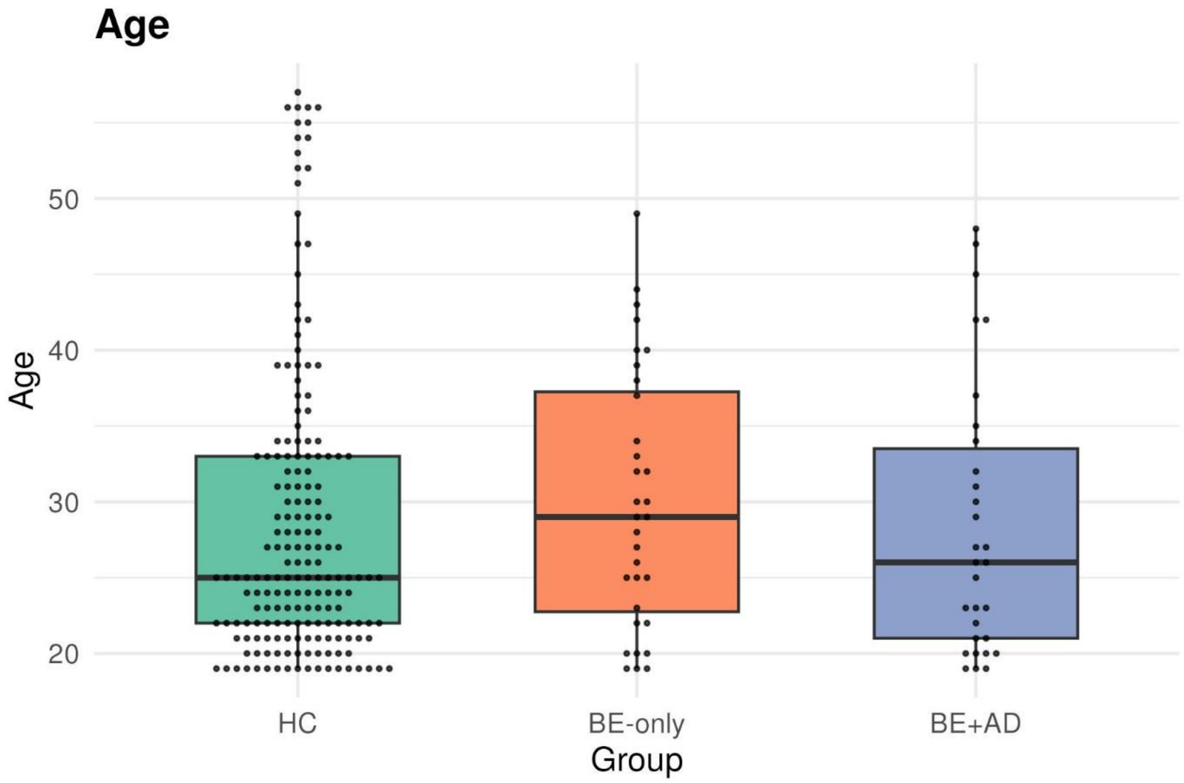

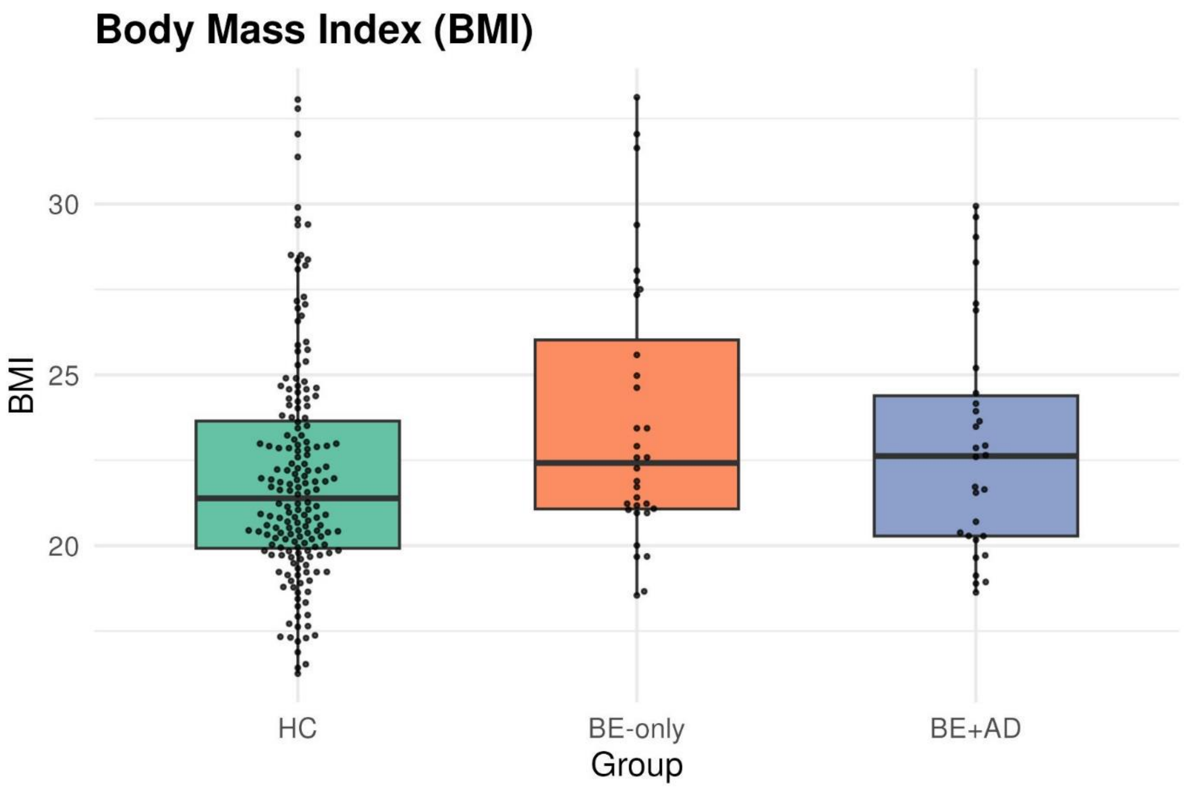
Body Mass Index (BMI)
30
25
20
BMI
HC
BE-only
BE+AD
Group

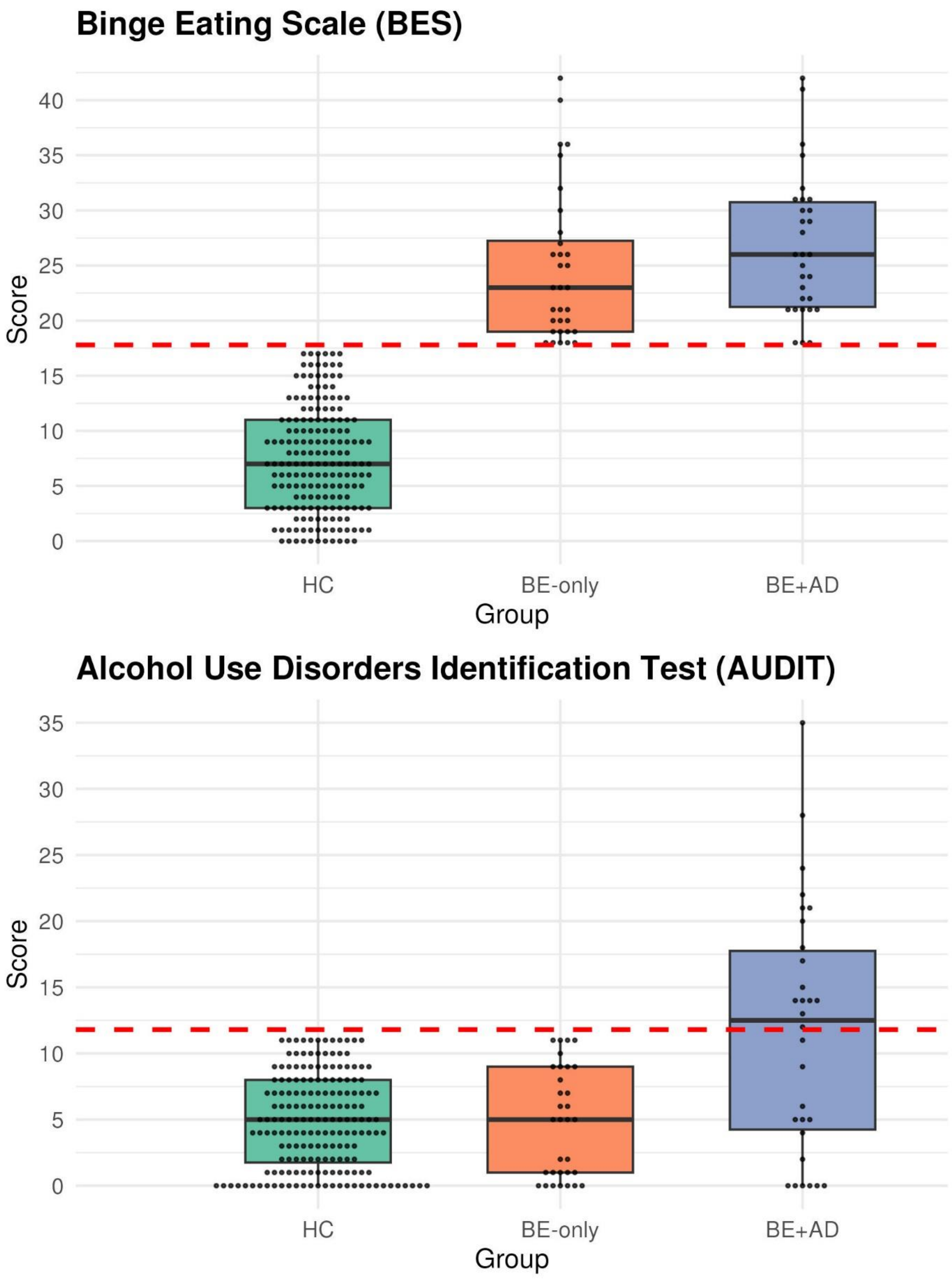
Binge Eating Scale (BES)
Score
0
5
10
15
20
25
30
35
40
HC
BE-only
BE+AD
Group
Alcohol Use Disorders Identification Test (AUDIT)
Score
0
5
10
15
20
25
30
35
HC
BE-only
BE+AD
Group

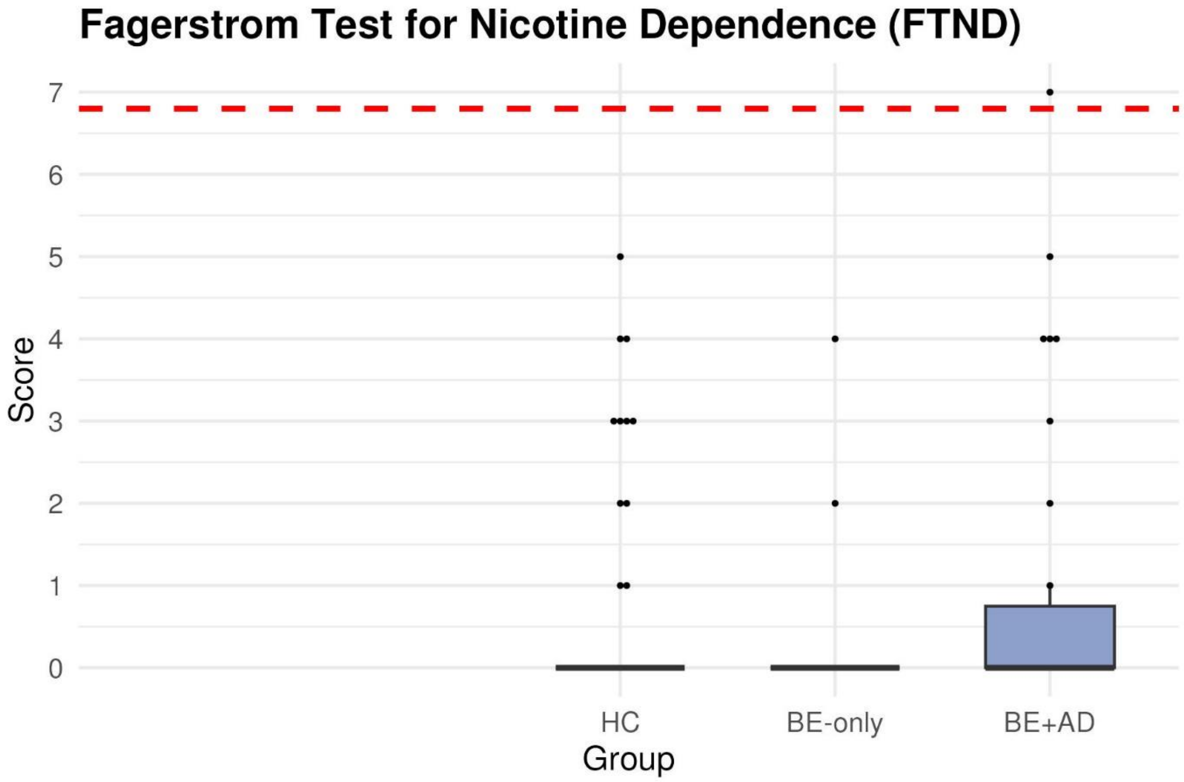
Fagerstrom Test for Nicotine Dependence (FTND)
Score
0
1
2
3
4
5
6
7
HC
BE-only
BE+AD
Group

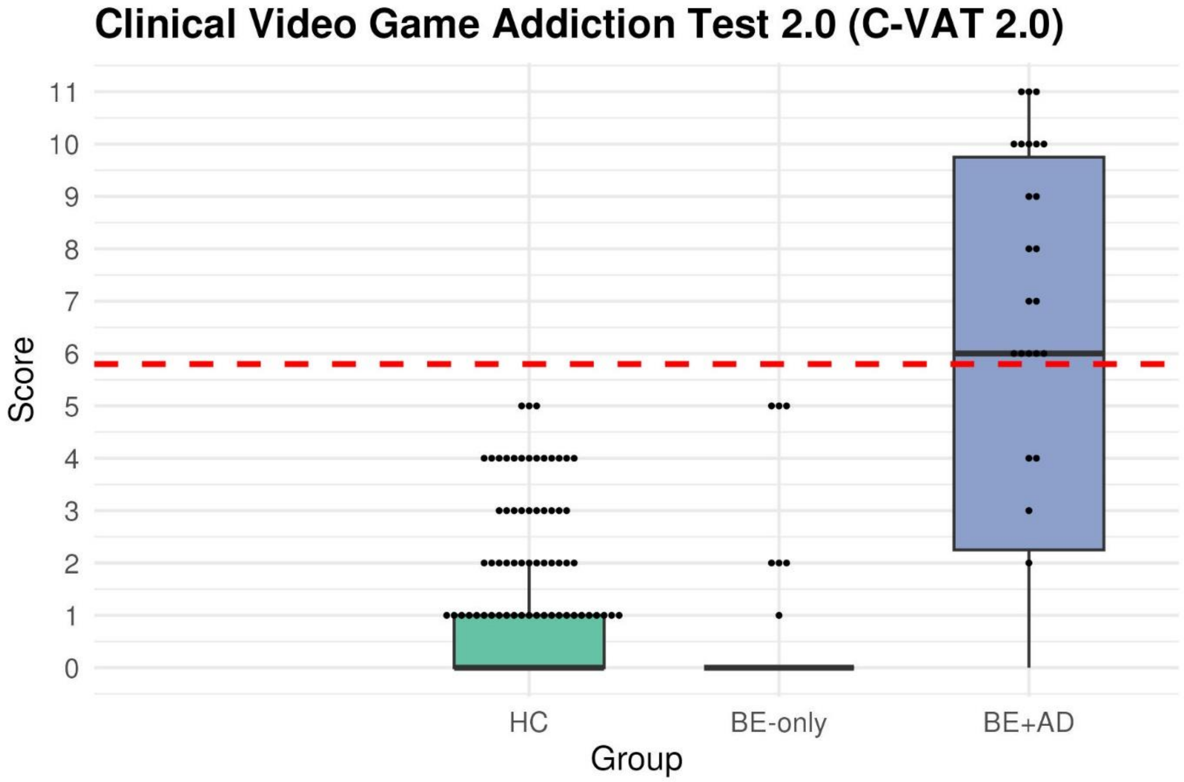
Clinical Video Game Addiction Test 2.0 (C-VAT 2.0)
Score
0
1
2
3
4
5
6
7
8
9
10
11
HC
BE-only
BE+AD
Group

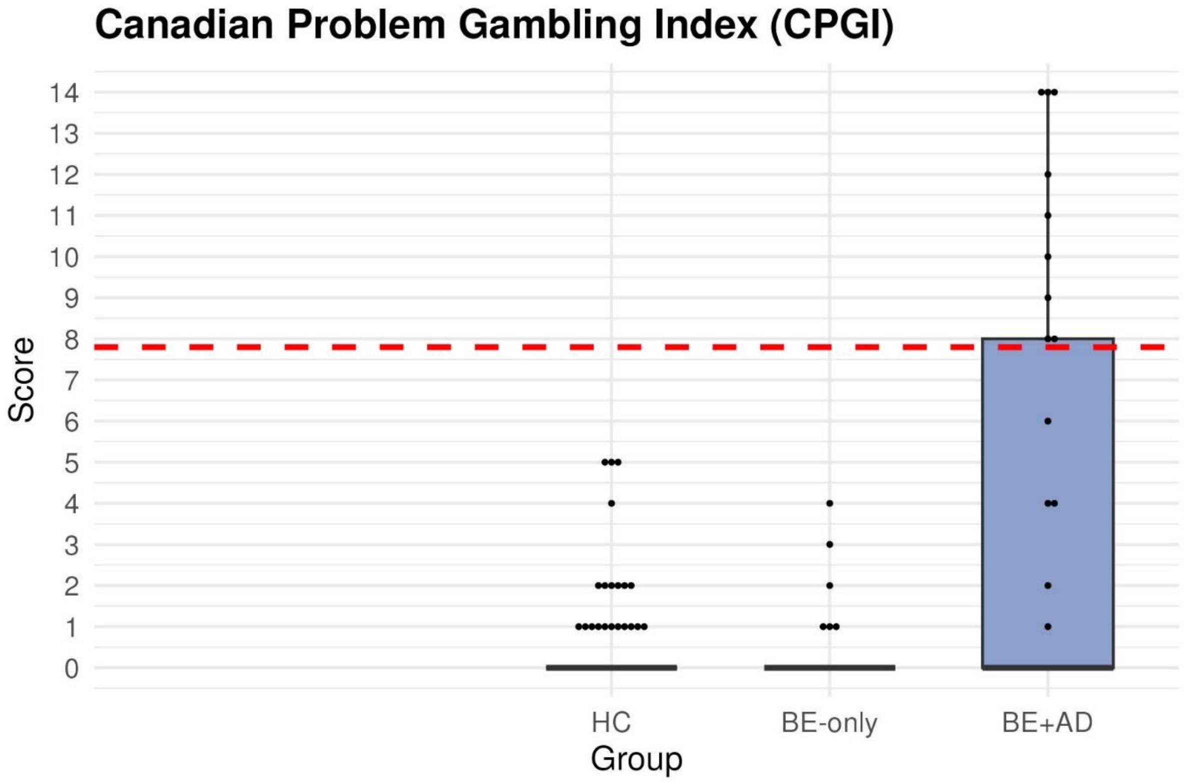
Canadian Problem Gambling Index (CPGI)
Score
14
13
12
11
10
9
8
7
6
5
4
3
2
1
0
HC
BE-only
BE+AD
Group

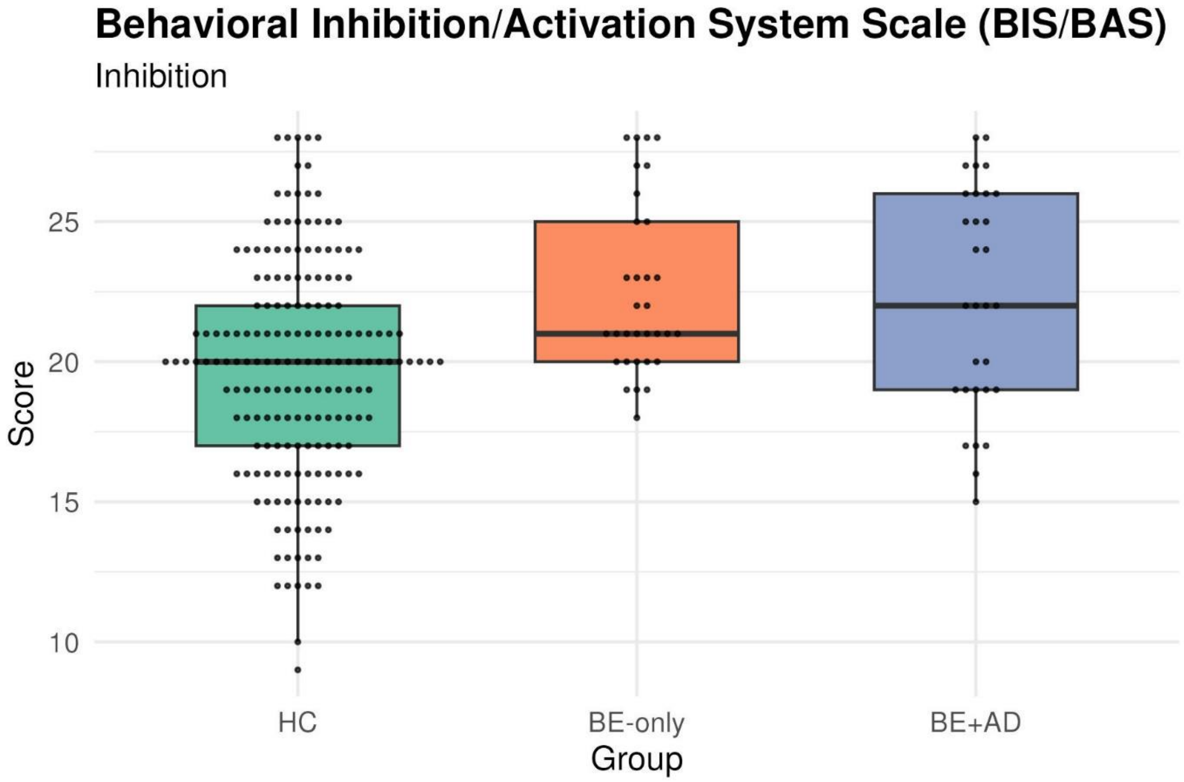
Behavioral Inhibition/Activation System Scale (BIS/BAS)
Inhibition
Score
25
20
15
10
HC
BE-only
BE+AD
Group

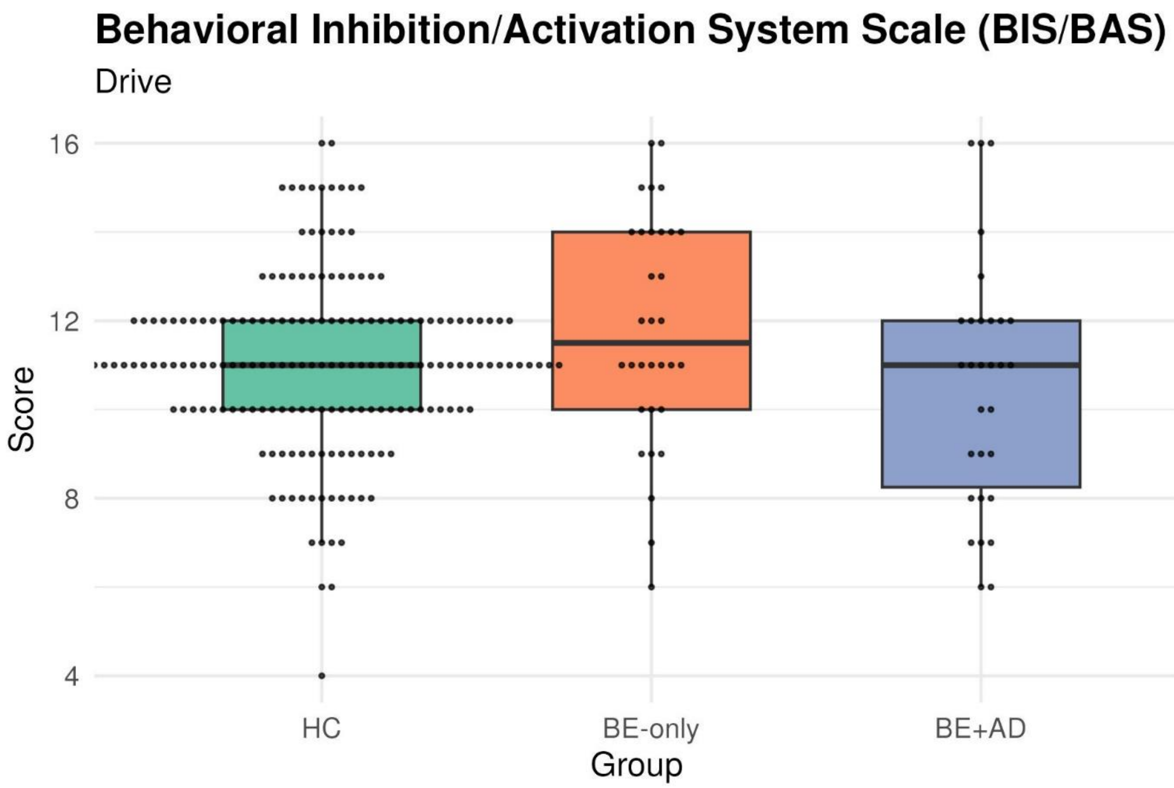
Behavioral Inhibition/Activation System Scale (BIS/BAS)
Drive
16
12
8
4
Score
HC
BE-only
BE+AD
Group

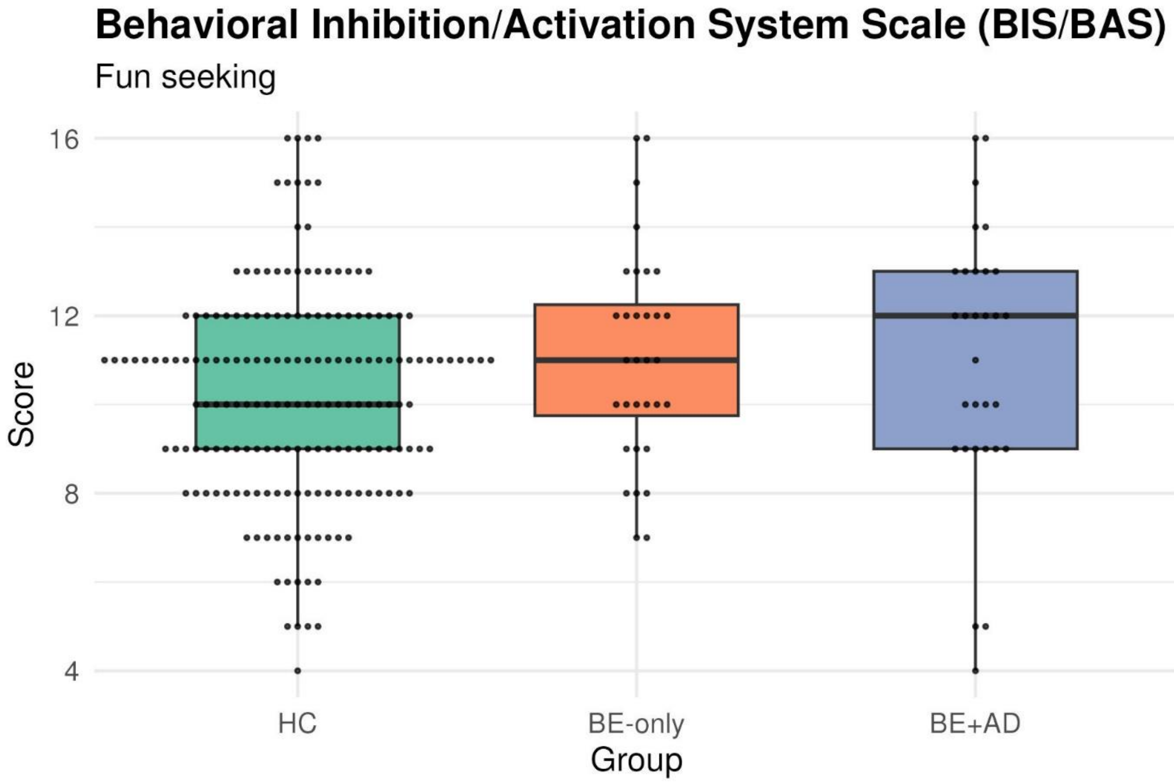
Behavioral Inhibition/Activation System Scale (BIS/BAS)
Fun seeking
16
12
8
4
Score
HC
BE-only
BE+AD
Group

Behavioral Inhibition/Activation System Scale (BIS/BAS)

Reward responsiveness

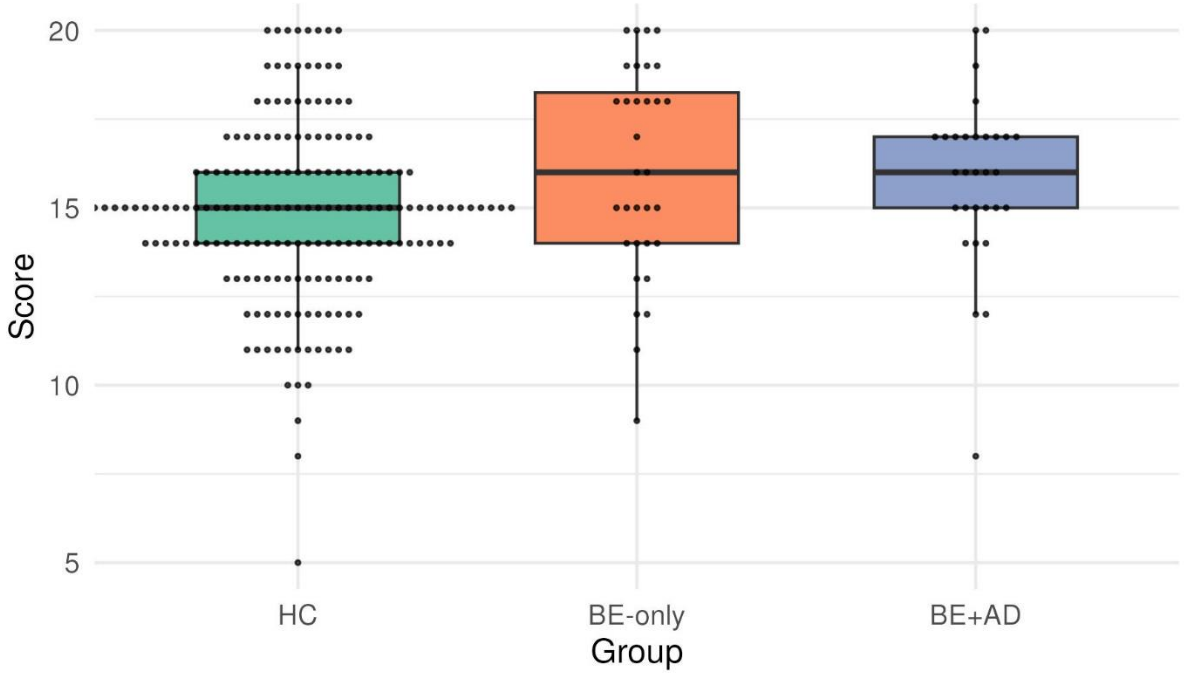

Barratt Impulsiveness Scale-11 (BIS-11)

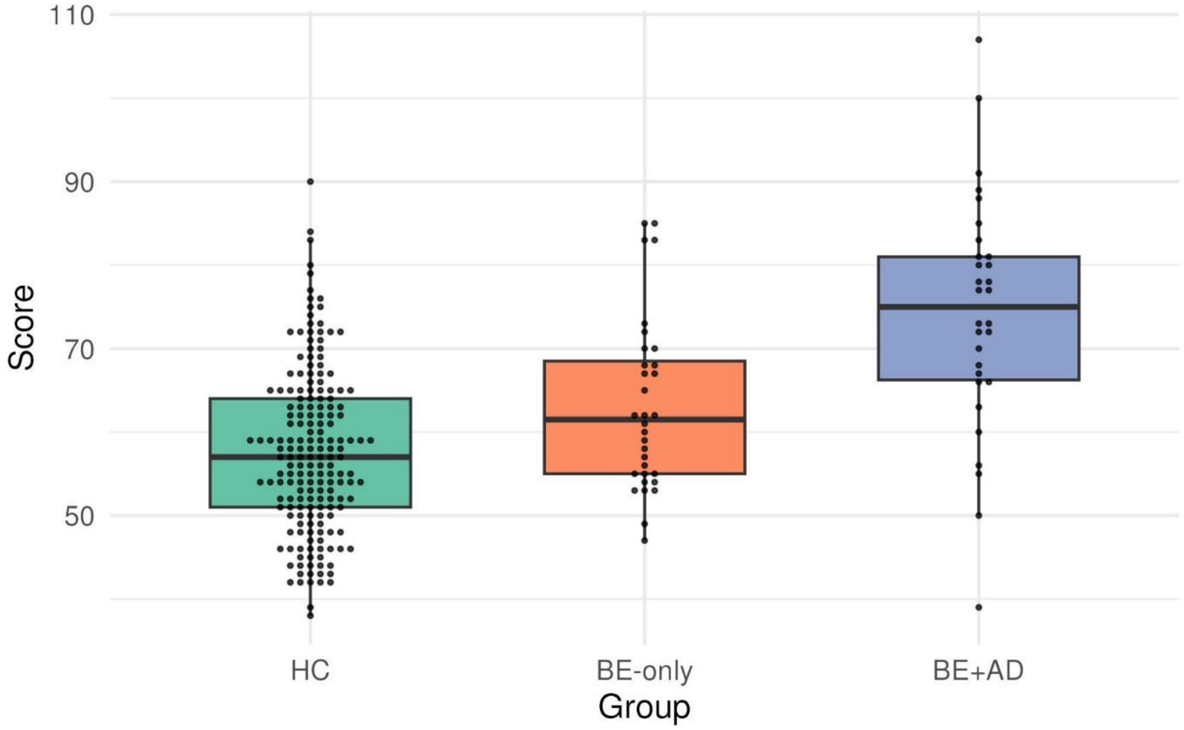

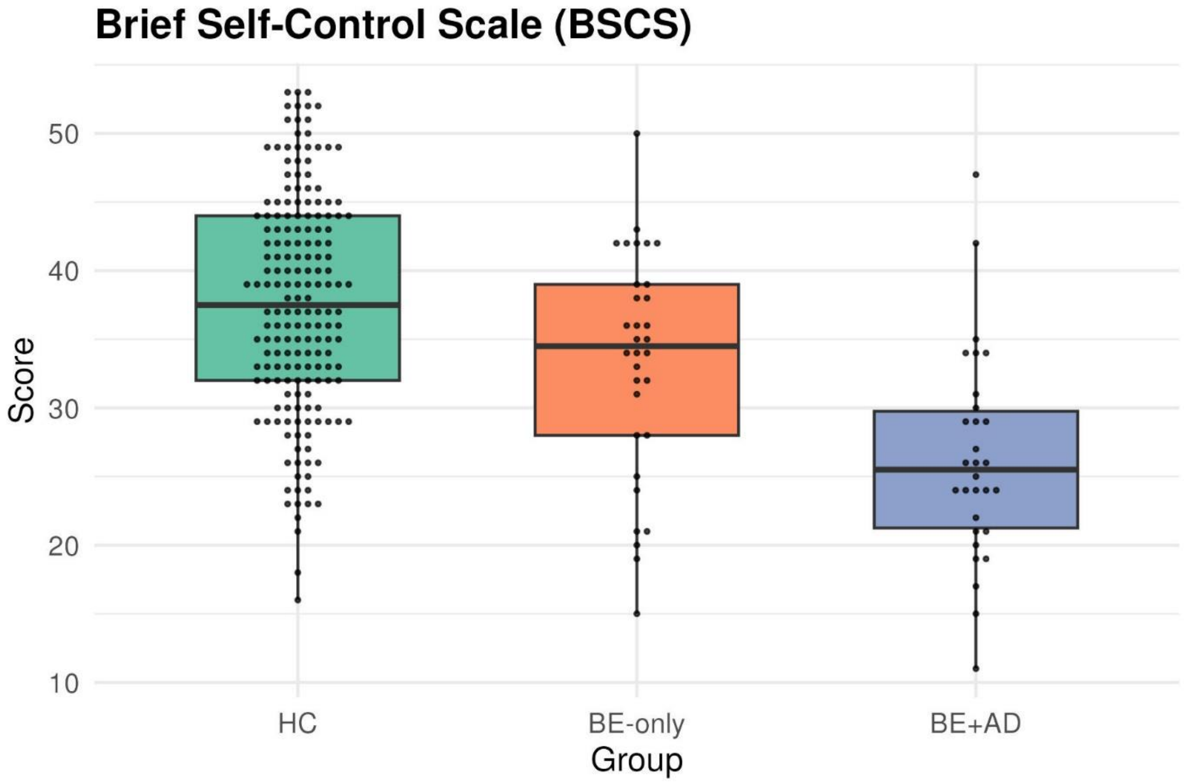
Brief Self-Control Scale (BSCS)
Score
50
40
30
20
10
HC
BE-only
BE+AD
Group

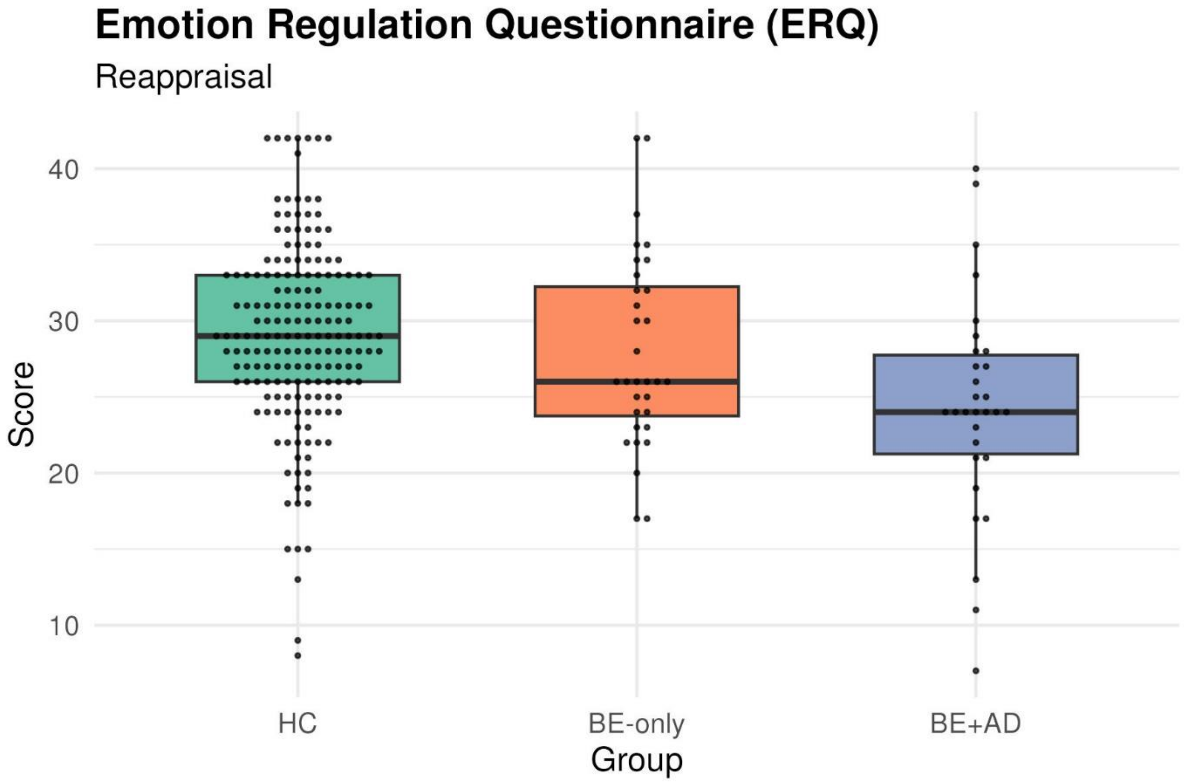
Emotion Regulation Questionnaire (ERQ)
Reappraisal
Score
40
30
20
10
HC
BE-only
BE+AD
Group

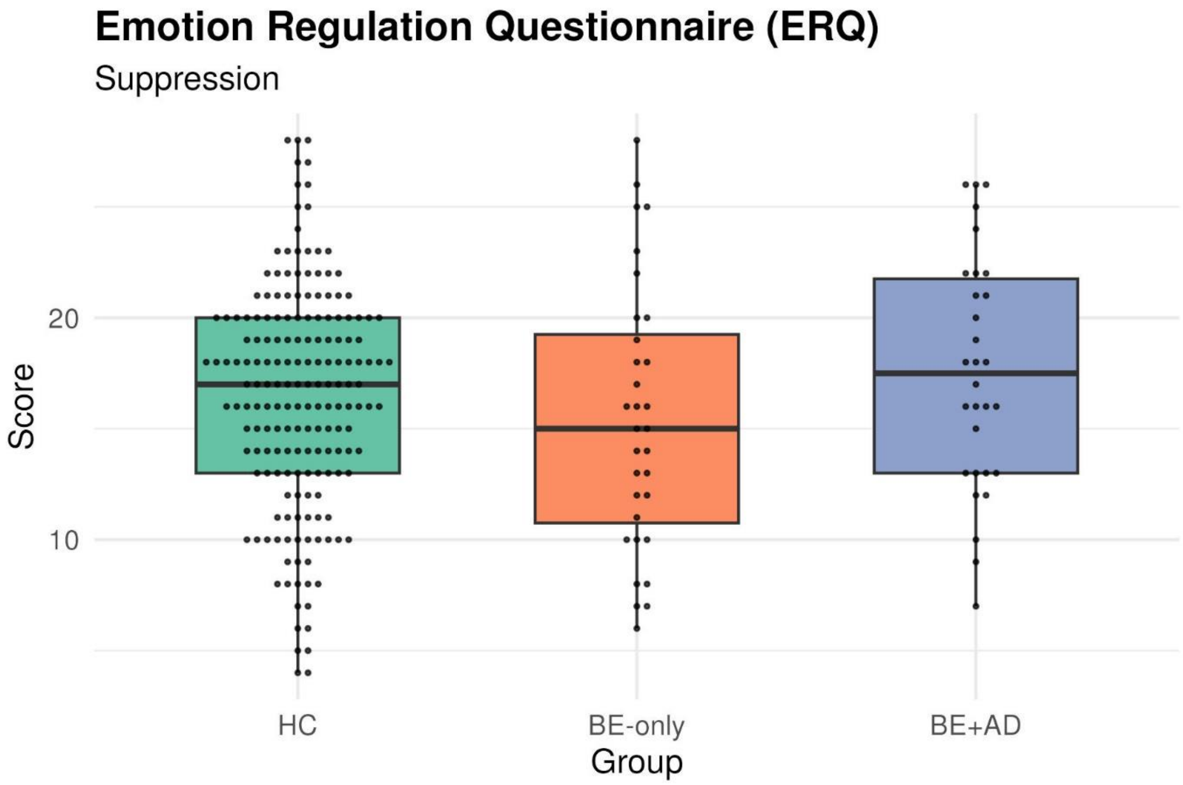
Emotion Regulation Questionnaire (ERQ)
Suppression
Score
20
10
HC
BE-only
BE+AD
Group

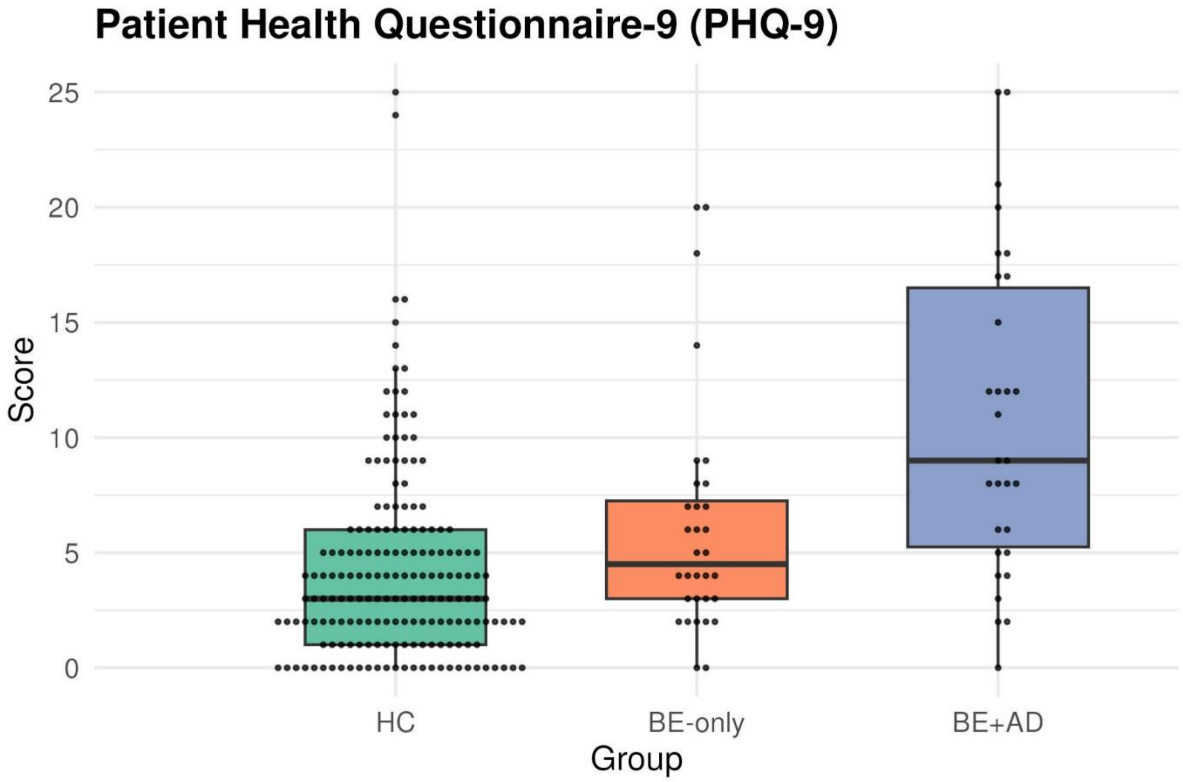
Patient Health Questionnaire-9 (PHQ-9)
Score
25
20
15
10
5
0
HC
BE-only
BE+AD
Group

Perceived Stress Scale (PSS)

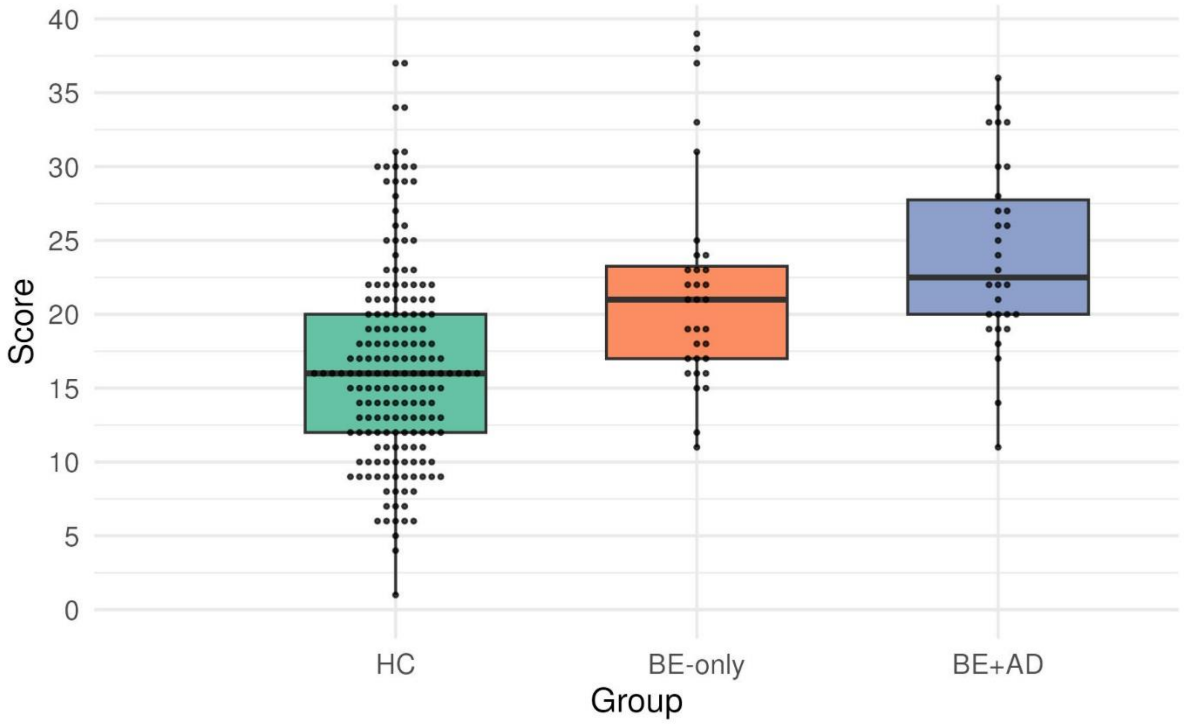

State/Trait Anxiety Inventory-X (STAI-X)

State Anxiety

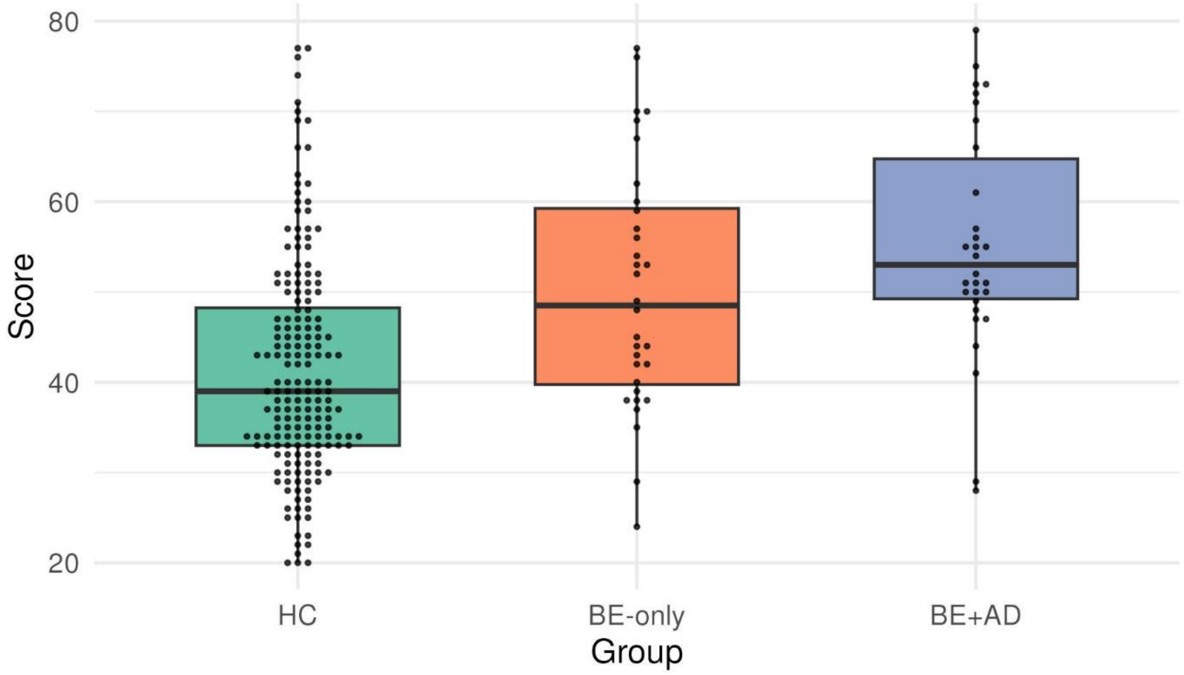

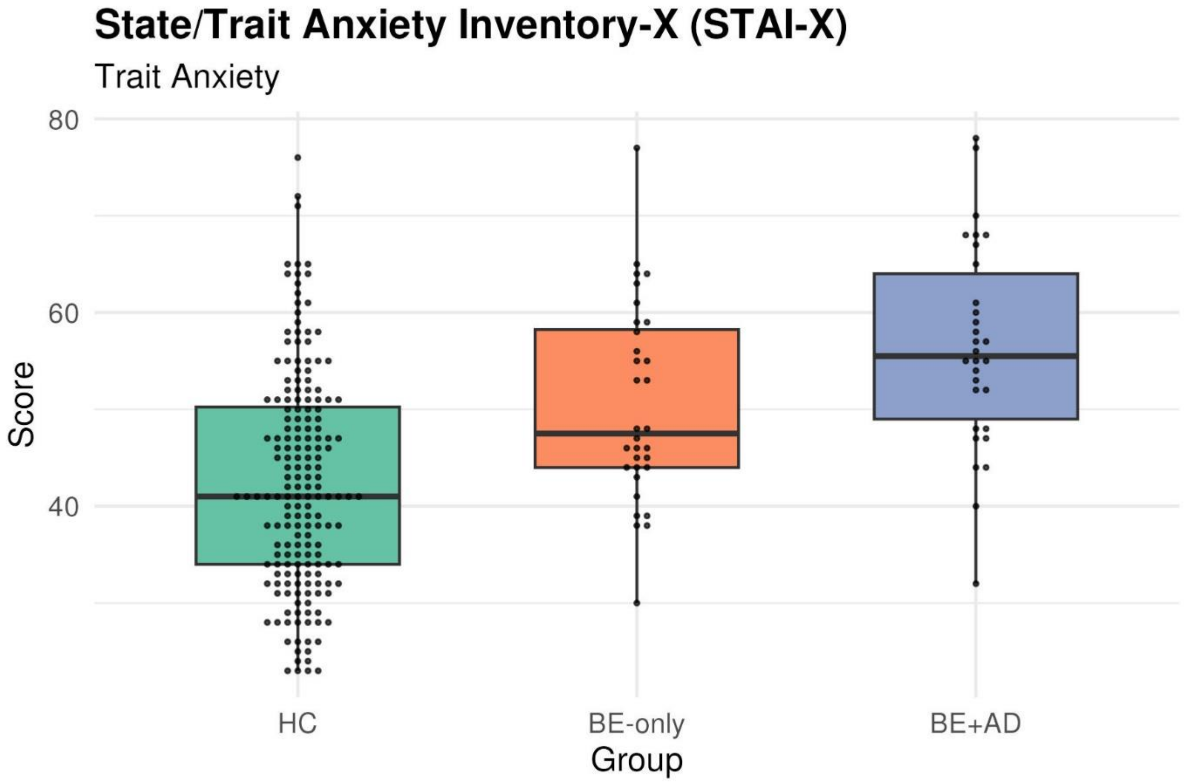

State/Trait Anxiety Inventory-X (STAI-X)
Trait Anxiety
80
60
40
Score
HC
BE-only
BE+AD
Group
